# A diet-microbial metabolism feedforward loop modulates intestinal stem cell renewal in the stressed gut

**DOI:** 10.1038/s41467-020-20673-4

**Published:** 2021-01-11

**Authors:** Yuanlong Hou, Wei Wei, Xiaojing Guan, Yali Liu, Gaorui Bian, Dandan He, Qilin Fan, Xiaoying Cai, Youying Zhang, Guangji Wang, Xiao Zheng, Haiping Hao

**Affiliations:** 1 State Key Laboratory of Natural Medicines, Jiangsu Province Key Laboratory of Drug Metabolism, China Pharmaceutical University, 210009 Nanjing, Jiangsu China; 2 Department of Pharmacology, School of Pharmacy, China Pharmaceutical University, 210009 Nanjing, Jiangsu China; 3 Department of Pharmacy, Shenzhen Luohu People’s Hospital, No. 47 Youyi Road, 518000 Shenzhen, China; 4 Tianyi Health Sciences Institute (Zhenjiang), 212000 Zhenjiang, Jiangsu China; 5 School of Basic Medicine and Clinical Pharmacy, China Pharmaceutical University, 210009 Nanjing, Jiangsu China

**Keywords:** Microbiome, Inflammatory bowel disease

## Abstract

Dietary patterns and psychosocial factors, ubiquitous part of modern lifestyle, critically shape the gut microbiota and human health. However, it remains obscure how dietary and psychosocial inputs coordinately modulate the gut microbiota and host impact. Here, we show that dietary raffinose metabolism to fructose couples stress-induced gut microbial remodeling to intestinal stem cells (ISC) renewal and epithelial homeostasis. Chow diet (CD) and purified diet (PD) confer distinct vulnerability to gut epithelial injury, microbial alternation and ISC dysfunction in chronically restrained mice. CD preferably enriches *Lactobacillus reuteri*, and its colonization is sufficient to rescue stress-triggered epithelial injury. Mechanistically, dietary raffinose sustains *Lactobacillus reuteri* growth, which in turn metabolizes raffinose to fructose and thereby constituting a feedforward metabolic loop favoring ISC maintenance during stress. Fructose augments and engages glycolysis to fuel ISC proliferation. Our data reveal a diet-stress interplay that dictates microbial metabolism-shaped ISC turnover and is exploitable for alleviating gut disorders.

## Abstract (web summary)

Here, using a mouse model of chronic stress, the authors investigate how diet impacts stress and gut microbial structure and epithelial integrity and show that dietary raffinose metabolism to fructose couples stress-induced gut microbial remodeling to intestinal stem cell renewal and epithelial homeostasis.

## Introduction

The gut microbiota has an intimate link with host health and disease. As an essential basis of this crosstalk, gut microbial ecology and function are exquisitely sensitive to a large array of host-derived cues typically diet and drugs^1–3^. In addition, a close link also exists between gut microbial structure and psychosocial inputs such as social stress^4,5^. Although not fully understood, this collection of microorganisms are dynamically engaged with niche signals such as gut lumen acidity, redox potential, and epithelial energetics to achieve symbiosis and adaption to host signals^6^. Deciphering the highly orchestrated and dynamic nature of gut microbe remodeling and the causal links with host pathophysiology is a fundamental task before harnessing them to improve host health.

The gut microbiota is naturally exposed to multiple remodeling factors from host daily life. However, little is known about the intricate interactions of co-existing host inputs in shaping the gut commensals and thereby host pathophysiology^3,7^. For example, although both dietary pattern and psychological stressors are separately known to drive gut microbial changes, how these ubiquitous lifestyle factors coordinately reshape the gut ecosystem is obscure. This is an important knowledge gap given that the gut microbiota might be a central hub in integrating multiple environmental factors, which translates to host responses at local and distant sites^8–10^. It is thus imperative to better understand host-microbe interplay in a broader context of interconnective remodeling signals.

Gastrointestinal (GI) disorders such as inflammatory bowel disease have become a global health issue inextricably linked to the dietary structure and psychosocial burden in modern society^11,12^. To date, a plethora of studies has advanced our understanding of these risk factors, focusing on separate impacts on the gut microbiota^13,14^. Typically, a recent study formulating more than 40 unique diets showed that gut microbial density is sensitive to the perturbation of dietary nutrients, with the concentration of protein and fiber having the strongest effect on mucosal inflammation and colitis development^15^. However, precisely how dietary and psychological factors interplay in remodeling the gut microbiota and affecting gut pathophysiology is yet to be answered. In this study, we aim to address the fundamental question of how diet meets with stress to dictate the outcome of gut epithelial disturbance. In a mice model of chronic restraint stress (RS), which is validated to have gut epithelial dysfunction^16,17^, we observe that chow diet (CD) or purified diet (PD), which differs in multiple ingredients including dietary fibers and amino acids, markedly modifies the impact of psychological stress on gut microbial structure and epithelial integrity. Using metagenomic profiling and gnotobiotic mice, we identify a feedforward metabolic loop between dietary raffinose and *Lactobacillus* that is mechanistically linked with intestinal stem cell (ISC) renewal and epithelial repair during stress. We further demonstrate the possibility of raffinose/fructose-based dietary manipulations to combat the detrimental impact of psychological stress on ISC maintenance and GI homeostasis.

## Results

### Dietary pattern dictates gut epithelial vulnerability to chronic stress

**Fig. 1:**
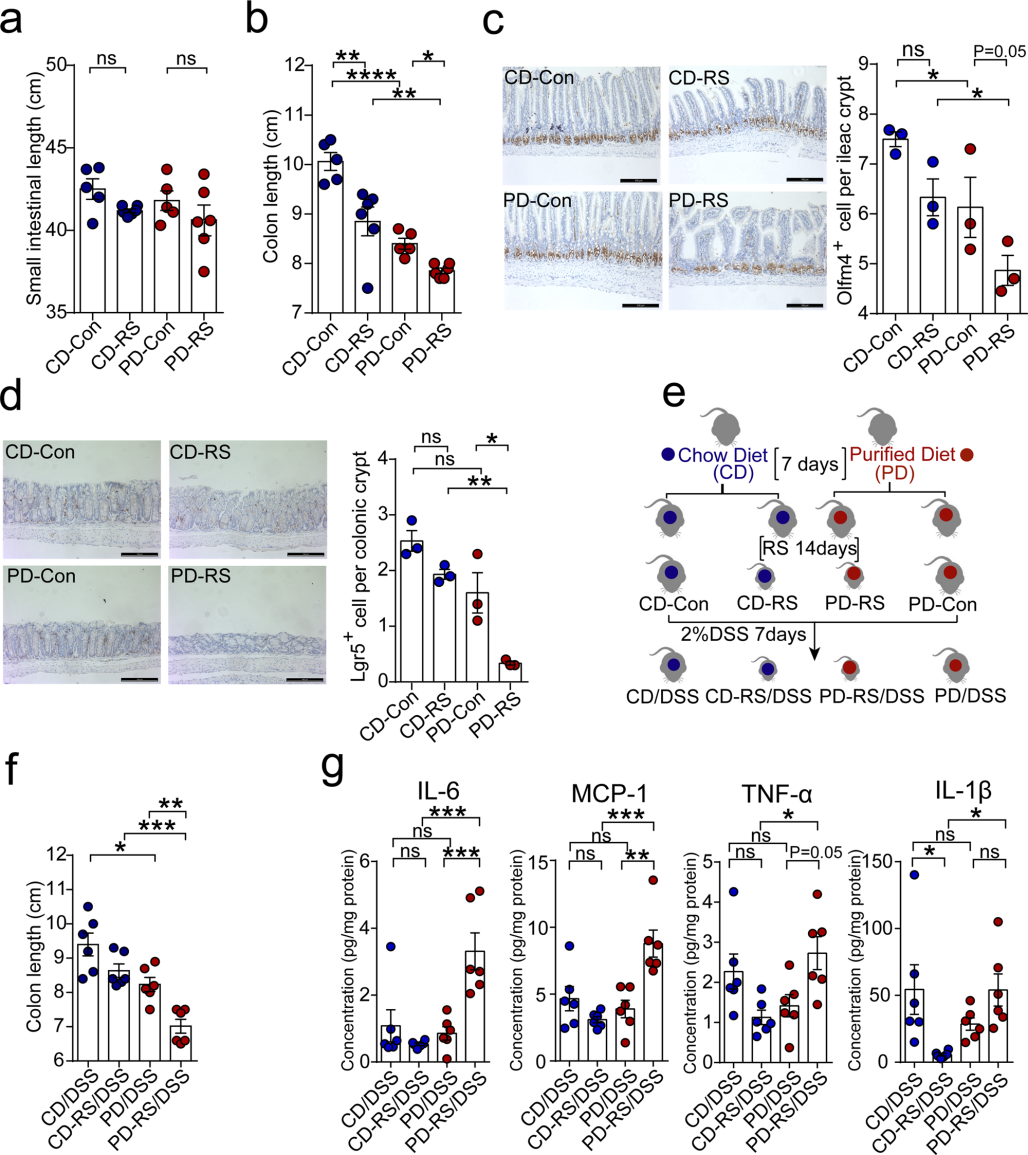
Diet-dependent difference in gut epithelial vulnerability to chronic stress. **a**, **b** The length of the small intestine (**a**) and colon (**b**) of mice after 14 days of restraint stress (RS, 4 h daily). Data are represented as mean ± SEM for *n* = 5 for the control group and *n* = 6 for the stress group, respectively. CD chow diet, PD purified diet. **c** Representative Olfm4 staining images in the ileum section of the intestine. Olfm4-positive cells are quantified at the bottom of per crypt. Scale bar: 100 µm. Data are represented as mean ± SEM for *n* = 3 (CD-Con vs PD-Con, *p* = 0.03; CD-RS vs PD-RS, *p* = 0.02; PD-Con vs PD-RS, *p* = 0.05). **d** Representative Lgr5 staining images in the colon. Lgr5-positive cells are quantified at the bottom of per crypt. Scale bar: 100 µm. Data are represented as mean ± SEM for *n* = 3 (CD-RS vs PD-RS, *p* = 0.002; PD-Con vs PD-RS, *p* = 0.01). **e** Schematic of the experimental design. Mice fed with either CD or PD were subjected to chronic RS for 14 days, and subsequently exposed to 2% (wt/vol) DSS for 7 days. **f** Colon length in colitis mice. Data are representative of three independent experiments and expressed as average length ± SEM for *n* = 6 (CD/DSS vs PD/DSS, *p* = 0.013; CD-RS/DSS vs PD-RS/DSS, *p* = 0.0007; PD-DSS vs PD-RS/DSS, *p* = 0.009). **g** ELISA assay of IL-1β, IL-6, TNF-α, and Mcp-1 in the colon tissue of colitis mice. Data are represented as average fold change ± SEM for *n* = 6. Statistical significance was determined by one-way ANOVA followed by Tukey’s post hoc test (**a**, **b**, **d**, **f**, **g**) or Fisher’s LSD test (**c**). *p < 0.05, **p < 0.01, ***p < 0.001, ****p < 0.0001; ns no significance.

RS in mice well recapitulates the detrimental effects of chronic stress exposure on gut homeostasis^18^. However, it is unclear whether the impact of stress is modulated by dietary alternation. To dissect potential dietary effects, mice were placed on either a rodent CD or PD, and subjected to a total of 14 consecutive days of RS (4 h daily) under either diet. The intestinal and colonic changes in morphology and histology of RS mice were examined afterward. Colonic, but not small intestinal, shortening was observed in stressed mice, with PD-fed mice exhibiting more pronounced change compared to the CD counterparts (Fig. 1a, b and Supplementary Fig. 1a). The histopathological examination further revealed a reduction in ileac villi length in CD and PD-fed mice after stress (Supplementary Fig. 1b). Moreover, PD exposure was associated with an aggravated decrease of ileac villi and colonic crypt size after stress, indicative of heightened stress susceptibility under PD (Supplementary Fig. 1b, c). The crypt is the niche of stem cells that undergo programmed proliferation and differentiation for the maintenance of epithelial integrity and function^19^. The reduced crypt size and villi length upon stress challenge prompted us to ask whether stress disturbed the proliferation of ISCs. As expected, RS induced a visible loss of stem cell density in the ileac (Olfm4-positive) and colonic (Lgr5-positive) crypts (Fig. 1c, d). We noticed that, in comparison with CD-fed counterparts, PD-fed mice showed a more drastic loss of stem cells in the ileum and distal colon section after stress (Fig. 1c, d). Moreover, PD was uniquely associated with a substantial reduction in Alcian blue-positive goblet cells in the colon (Supplementary Fig. 1d,e) and Lysozyme-positive Paneth cells in the intestine of stressed mice (Supplementary Fig. 1f), supporting a critical role of dietary pattern in shaping the proliferation and differentiation response of gut stem cells under conditions of chronic psychological stress.

We reasoned that the distinct impact of CD and PD on stress-induced disturbance of ISC proliferation and differentiation might translate to differential susceptibility to epithelial injury. To test this, after 14-day RS, a cohort of mice fed CD or PD received 2.0% DSS in drinking water for 7 days to induce inflammatory damage to gut epithelial cells (Fig. 1e). In mice fed on PD, RS exposure for 14 days markedly worsened colitis symptoms afterward, as shown by colonic shortening (Fig. 1f), histology (Supplementary Fig. 2a), loss of goblet cells (Supplementary Fig. 2b), and inflammatory cytokines (IL-6, MCP-1, TNF-α; Fig. 1g). In sharp contrast, stressed mice maintained on CD were much less susceptible to DSS-induced epithelial damage and inflammatory injury (Fig. 1f, g and Supplementary Fig. 2a). We further confirmed that, when mice were exposed to chemical insults during the last 7 days of stress exposure (Supplementary Fig. 2c), CD and PD-fed mice still differed strikingly in parameters of gut epithelial injury after stress, as evidenced by disease activity index (DAI), colon length, tissue pathology and cytokine profiles (Supplementary Fig. 2d–g). Overall, these data suggest that the deteriorating effect of psychological stress on gut epithelium is subjected to dietary modification, potentially explained by distinct responses of stem cell maintenance.

### Diet interplays with stress in remodeling the gut microbiota

**Fig. 2:**
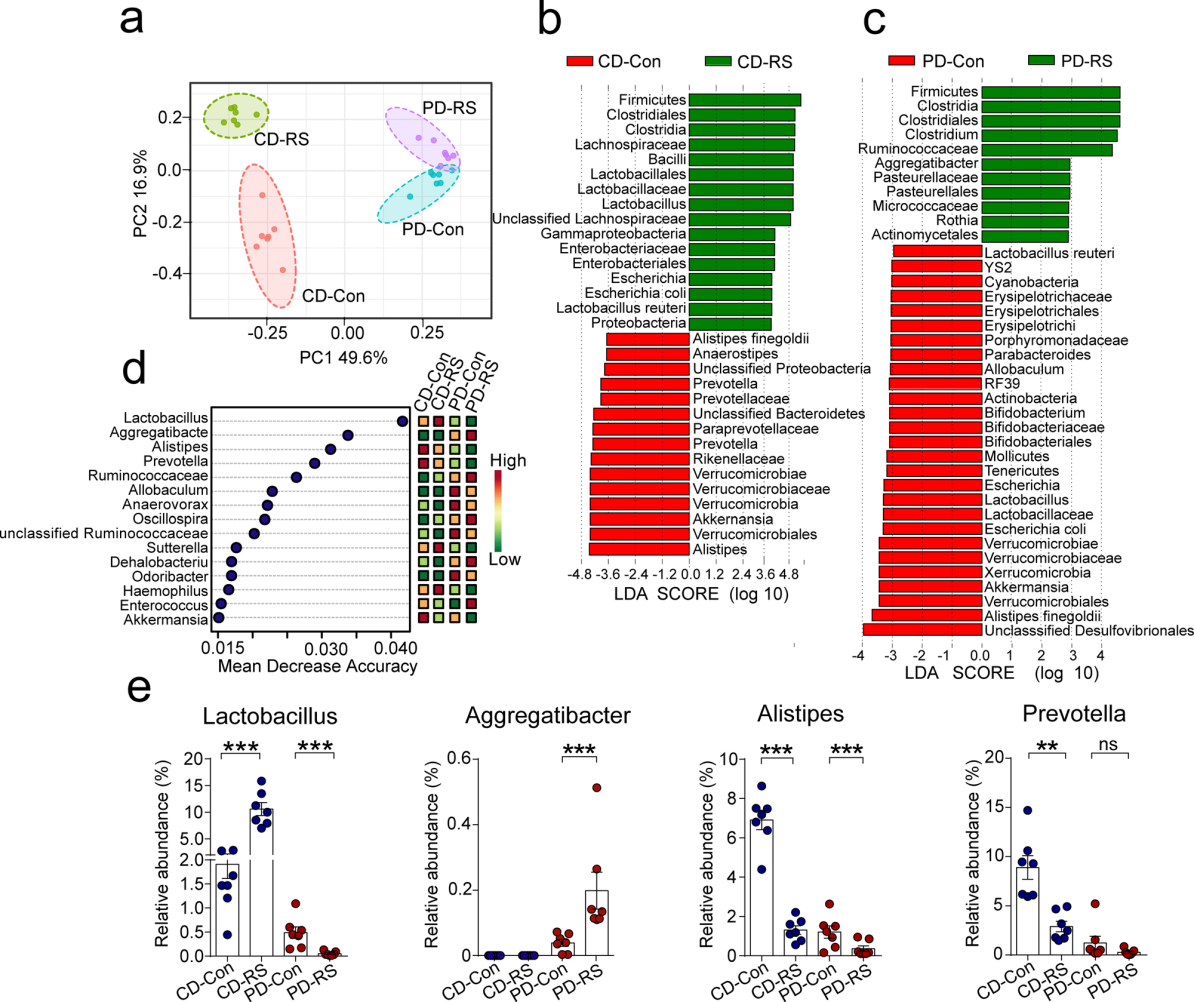
Diet interplays with stress in remodeling the gut microbiota. **a** PCoA plot of bacterial Operational Taxonomic Unit (OTU) in fecal samples of mice fed with chow diet (CD) or purified diet (PD) at the end of 14-day restraint stress (RS). The community distance is based on Bray–Curtis dissimilarity metrics of relative abundance determined by 16s rRNA sequencing. Each symbol represents the data of an individual mouse (*n* = 7). **b**, **c** Linear discriminative analysis (LDA) score of differentially expressed bacteria obtained from LEfSe analysis of fecal microbiota in mice fed with CD diet (**b**) or PD (**c**). A genus with Kruskal–Wallis ≤ 0.05, as well as LDA > 3.6 (**b**) or 3 (**c**) are shown. **d** The top 15 distinct bacterial genera identified by applying Random Forest Regression analysis of their relative abundances in control and stressed mice. **e** The relative abundance of *Lactobacillus*, *Aggregatibacter*, *Alistipes*, and *Prevotella* in the fecal microbiota of mice. Data are presented as mean ± SEM for *n* = 7. **p < 0.01 and ***p < 0.001 by Wilcoxon rank-sum test. ns no significance.

Diet and psychological stress alone are known to change the gut microbiota, but it is unclear how these two ubiquitous factors coordinately dictate the gut microbial landscape. To query how dietary patterns impact gut microbiome remodeling under psychological stress, 16S ribosomal RNA (rRNA) gene sequencing of fecal pellets from CD or PD-fed mice was performed at the end of 14-day RS (Supplementary Fig. 3a). Stress induced a slight but significant decrease in the alpha diversity of gut microbiota in mice fed PD but not CD (Supplementary Fig. 3b). When beta-diversity was analyzed using the Bray–Curtis Dissimilarity metric in principle coordinate analysis (PCoA), we observed a clear segregation of gut microbial signatures between CD and PD groups (Fig. 2a). LDA effect size (LEfSe) analysis of the taxonomic alternations revealed that stress elicited unique sets of bacterial enrichment under the two dietary patterns. In particular, stress-enriched bacteria in mice fed with CD largely belonged to the *Lactobacillales*, *Enterobacteriales,* and *Lachnospiraceae* (Fig. 2b and Supplementary Fig. 3c), whereas *Pasteurellales*, *Actinomycetales,* and *Ruminococcaceae* typically bloomed when stressed mice were maintained on a PD (Fig. 2c and Supplementary Fig. 3d). A random forest analysis revealed that the microbial genus that showed the strongest impact on accuracy belonged to the *Lactobacillus spp*. (Fig. 2d), which were remarkably enriched in CD-fed mice after stress but substantially depleted under PD (Fig. 2e). In contrast, other genera that topped on this analysis, including *Aggregatibacter*, *Alistipes,* and *Prevotella*, did not show such a conversing trend of change under the two diets (Fig. 2e). These findings collectively suggest a strong impact of the dietary shift on stress-induced gut microbial remodeling, notably represented by the change of *Lactobacillus* abundance.

To test whether such a diet-dependent microbial remodeling could also be witnessed under inflammatory conditions, we further determined the fecal microbiota composition at the 10th day of DSS exposure after RS. As expected, we found that *Lactobacillales* were among the top enriched genera in previously stressed mice fed on CD (Supplementary Fig. 3e), indicating a long-lasting impact of CD components on the abundance of *Lactobacillus* spp. Based on all these considerations, we reasoned that the *Lactobacillus* might serve as a key genus to mediate the effect of dietary shift on gut epithelial change.

### Chow diet-enriched *Lactobacillus* sustain epithelial renewal under stress

**Fig. 3:**
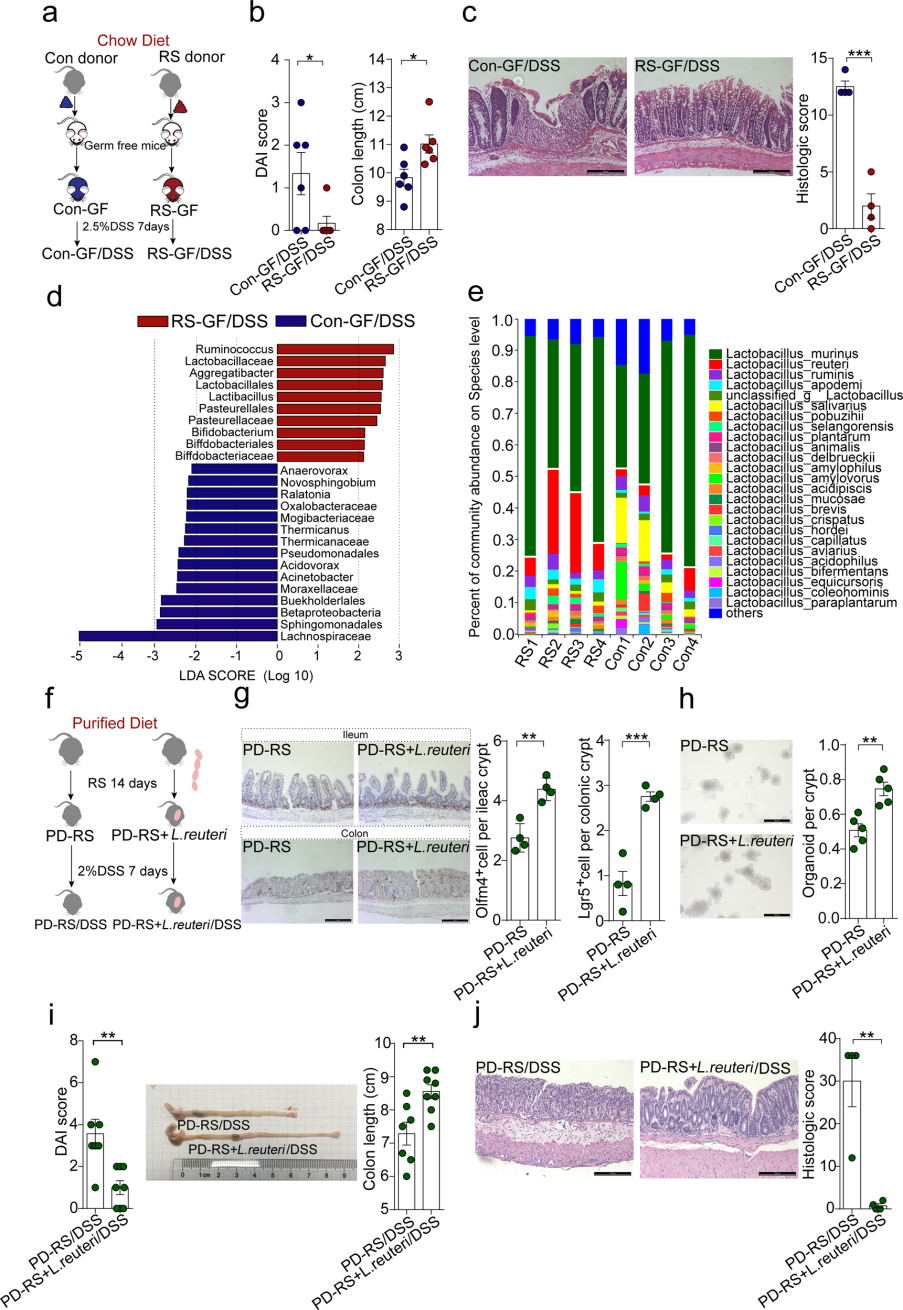
Diet-enriched *Lactobacillus* strains sustain epithelial renewal in stressed mice. **a** Schematic of the experimental design to assess the role of stress-shaped gut microbiota in epithelial injury. Germ-free (GF) mice received fecal microbiota transplant (FMT) derived from control (Con-GF) or restraint-stressed (RS-GF) donor mice fed chow diet. One week after FMT, mice were treated with 2.5% (wt/vol) DSS for 7 days. **b** Disease activity index (DAI) score and colon length of recipient mice. Data are represented as mean ± SEM for *n* = 6 (*p* = 0.04 for DAI; *p* = 0.02 for colon length). **c** Representative H&E staining images and a histologic score of distal colon section of mice. Scale bar: 100 µm. Data are represented as mean ± SEM for *n* = 4 (*p* = 0.0001). **d** Linear discriminative analysis (LDA) score obtained from LEfSe analysis of cecum microbiota in Con-GF and RS-GF mice with colitis. A genus with Kruskal–Wallis ≤ 0.05, as well as LDA > 2 is reported. **e** Average relative abundance of representative *Lactobacillus* species of chow diet-fed mice with or without stress. Data are represented as average relative abundance ± SEM for *n* = 4. **f** Schematic of the experimental design to assess the contribution of *Lactobacillus reuteri* (*L. reuteri*) in epithelial response to stress. Mice fed on a purified diet (PD) were colonized with live *L. reuteri* (2*10^8^ CFU per mouse) or heat-killed controls every day during 14 days of restraint stress. A cohort of mice was subsequently exposed to 2% (wt/vol) DSS for 7 days. **g** Representative Olfm4 staining images of the ileum (upper panel) and Lgr5 staining images of the colon (lower panel) from stressed mice fed with PD. Quantification was performed for positive cells per crypt. Scale bar: 100 µm. Data are representative of two independent experiments and expressed as mean ± SEM for n = 4 (*p* = 0.001 for Olfm4 staining; *p* = 0.0005 for Lgr5 staining). **h** Representative images of organoids derived from the intestinal crypts of restraint-stressed (RS) mice. Scale bar: 200 µm. Data are represented as Mean ± SEM for n = 5 (*p* = 0.0024). **i** DAI score and colon length of colitis mice with prior restraint stress for 14 days. Data are represented as mean ± SEM for *n* = 8 (*p* = 0.037 for DAI; *p* = 0.006 for colon length). **j** Representative H&E staining images of distal colon section from colitis mice with or without prior *L. reuteri* treatment. Scale bar: 100 µm. Data are represented as mean ± SEM for *n* = 4 biologically independent samples (*p* = 0.0028). Statistical significance was all determined by unpaired two-tailed student’s *t*-test, *p < 0.05, **p < 0.01, ***p < 0.001.

Our time-dependent analysis of fecal microbial shift of CD-fed mice during RS revealed a prompt and continuous change of gut microbial composition after stress, which was detectable on the 3rd day and reached the maximal divergence on the 14th day (Supplementary Fig. 4a). To further understand the causal relationship between stress-induced gut microbial alternations and epithelial response, we performed fecal microbiota transplant (FMT) from CD-fed control and stressed mice into germ-free (GF) recipients and triggered epithelial injury by DSS (Fig. 3a). GF mice transplanted with the fecal microbiota from stressed mice (RS-GF/DSS) showed attenuated colitis phenotypes including DAI, colon length, and crypt density (Fig. 3b, c), suggesting that stress-induced adaptive changes in gut microbiota exert a protective effect against chemical injury. Notably, 16S rRNA sequencing of the cecum microbiome from colitis mice confirmed that *Lactobacillus* was enriched in recipient mice of RS-donor (Fig. 3d).

Among the altered gut microbial species, we considered *Lactobacillus spp*. to be uniquely associated with epithelial response to stress. *Lactobacillus* enrichment was specifically observed with CD (Fig. 2b, d), and increased *Lactobacillus* abundance was consistently associated with less vulnerability to stress or chemical insults after stress (Fig. 3d and Supplementary Fig. 3e). In consistence of these observations, *Lactobacillus* was previously reported to be protective of intestinal epithelia, although the mechanistic link is elusive^20^. To gain in-depth insight into the role of *Lactobacillus* on epithelial homeostasis under psychological stress, we conducted metagenomic sequencing of the fecal microbiome in CD-fed mice. This species-level analysis of microbial community enabled us to observe more comprehensive changes in bacterial structure triggered by stress (Supplementary Fig. 4b). Typically, an extensive change of multiple *Lactobacillus* strains was observed, and, among them, *Lactobacillus reuteri* (*L. reuteri*) showed the most striking increase in abundance after stress (Fig. 3e and Supplementary Fig. 4c). For functional assessment, we isolated a *Lactobacillus* strain from the feces of a stressed mouse, which showed 93% similarity to two published *L. reuteri* genomes and was identified as *L. reuteri* DSM 20016 (Supplementary Fig. 4d). Daily oral gavage of PD-fed mice with *L. reuteri* DSM 20016 during RS effectively preserved Olfm4-positive stem cells in the ileum as well as the colon (Fig. 3f, g). When cultured in vitro, intestinal crypts from *L. reuteri*-treated mice displayed a higher capability of self-expansion into organoids (Fig. 3h). Reintroducing *L. reuteri* in these stressed mice also protected against the susceptibility to chemical injury afterward, as shown by significantly improved colitis symptoms (Fig. 3i, j). Thus, these findings reveal that CD-enriched *Lactobacillus* strains have a homeostatic role in epithelial response to stress and may underlie the diet-dependent epithelial responses to chemical insults.

### Raffinose underlies diet-dependent gut epithelial response to stress

**Fig. 4:**
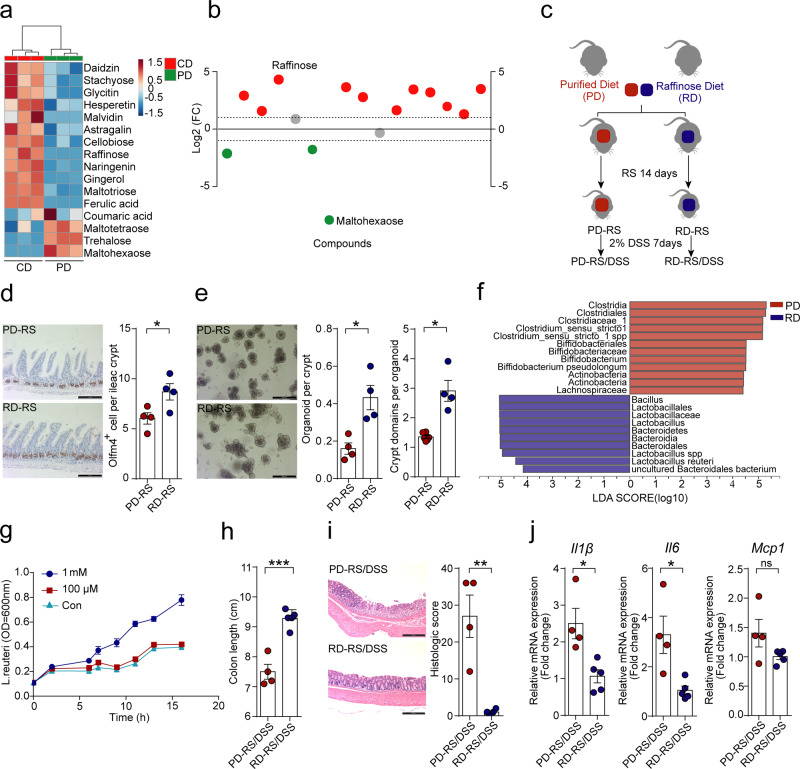
Raffinose underlies diet-dependent epithelial response to stress. **a** Heatmap showing the different abundance of oligosaccharides and polyphenolic compounds in chow diet (CD) and purified diet (PD). **b** Comparison of the Log2-fold changed abundance of oligosaccharides and polyphenolic acid components between CD and PD. Points in red and green represent components enriched in CD and PD, respectively. **c** Schematic of the experimental design to assess the role of dietary raffinose. Mice were fed with PD or PD supplemented with raffinose (RD, 100 g/kg diet) during 14 days of restraint stress (*n* = 10), and a cohort of mice was subsequently exposed to 2% (wt/vol) DSS for 7 days. **d** Representative Olfm4 staining images and quantification of Olfm4-positive cells per crypts in the ileum of mice after 14 days RS. Scale bar: 100 µm. Data are represented as mean ± SEM for *n* = 4 biologically independent samples (*p* = 0.03). **e** Representative images of organoids derived from the intestinal crypts of stressed mice fed on PD or RD. The  number of organoid per crypt and crypt domain per organoid were compared. Scale bar: 200 µm. Data are represented as mean ± SEM for *n* = 4 (*p* = 0.009 for organoid per crypt; *p* = 0.0006 for crypt domain per organoid). **f** Linear discriminative analysis (LDA) score obtained from LEfSe analysis of fecal microbiota in PD and RD-fed mice after stress. A genus with Kruskal–Wallis ≤ 0.05, as well as LDA > 4, are reported. **g** In vitro growth curve of *L. reuteri* with or without raffinose supplementation in the culture medium. Data are represented as the average optical density (OD, 600 nm) of culture ± SEM for *n* = 3. **h** Colon length of PD and RD-fed mice with DSS-induced colitis. Data are represented as mean ± SEM for *n* = 4 (*p* = 0.0002). **i** Representative H&E staining images and a histologic score of distal colon section of colitis mice. Scale bar: 200 µm. Data are represented as mean ± SEM for *n* = 4 (*p* = 0.004). **j** Relative mRNA expression of *Il1β*, *Il6*, and *Mcp1* in the colon tissue of colitis mice. Data are represented as average fold change ± SEM for PD-RS/DSS group (*n* = 4) and RD-RS/DSS group (*n* = 5). *p* = 0.01 for *Il1β*; *p* = 0.014 for *Il6*; *p* = 0.1 for *Mcp1*.Statistical significance in **d**, **e**, **h**–**j** was determined by unpaired two-tailed Student’s *t*-test. ns no significance. *p < 0.05, **p < 0.01, ***p < 0.001.

The CD and PD used in our study are isocaloric but differ in multiple ingredients (Supplementary Fig. 5a). This chemical divergence is characterized by the fact that cellulose and maltodextrin are abundant in PD, whereas CD is composed of various indigestible fibers from plant materials. Studies have shown that certain dietary components (e.g., oligosaccharides, non-digestible carbohydrates, and phenolic compounds) are highly associated with the bloom of *Lactobacillus* and *Bifidobacterium* species in the gut^21^. We thus hypothesized that CD components might provide a favorable condition to sustain the expansion of *Lactobacillus spp*. during stress. To identify the dominant food component that may explain the different gut microbial responses, we performed a thorough chemical analysis of the diets with special attention to oligosaccharide and polyphenol compounds. A total of 148 components reached significantly different abundance in CD (enriched with 127 components) and PD (Supplementary Fig. 5b), and, among them, 16 differential components belonged to oligosaccharides or polyphenol (Fig. 4a).

Raffinose, a dietary soluble fiber naturally occurring in vegetables^22^, is the most highly enriched component in CD but scarcely found in PD (Fig. 4b). To understand whether raffinose could underlie the diet-shaped epithelial response to stress, we fed the mice with raffinose-supplemented PD (RD) and examined the effects of RS on gut epithelial integrity (Fig. 4c). Compared with PD-fed mice, mice receiving RD had noticeably increased density of stem cells in the intestine (Olfm4-positive) and colon (Lgr5-positive) after stress (Fig. 4d and Supplementary Fig. 5c). RD also boosted the ability of intestinal crypts from stressed mice to form organoids in vitro (Fig. 4e). Of note, we found that supplementation with raffinose proved effective to rescue PD-aggravated loss of *L. reuteri* during stress (Fig. 4f). To test whether raffinose may directly promote the growth of *Lactobacillus*, we examined the growth rate of *L. reuteri* with raffinose treatment. We found that 1 mM raffinose was clearly effective to promote *L. reuteri* growth (Fig. 4g), which may partially explain the maintenance of *L. reuteri* abundance under CD.

We next investigated whether dietary supplementation with raffinose could recapitulate the effect of CD to increase the resilience to epithelial injury. In line with our expectation, mice provided with RD showed decreased colonic shortening and much less evidence of epithelial injury, crypt loss, and inflammatory disturbance, as compared to the PD counterparts (Fig. 4h–j). Together, these findings suggest that dietary raffinose abundance appears to be the major factor driving gut microbial and epithelial response to stress.

### Fructose produced from raffinose by *L. reuteri* promotes ISC proliferation

**Fig. 5:**
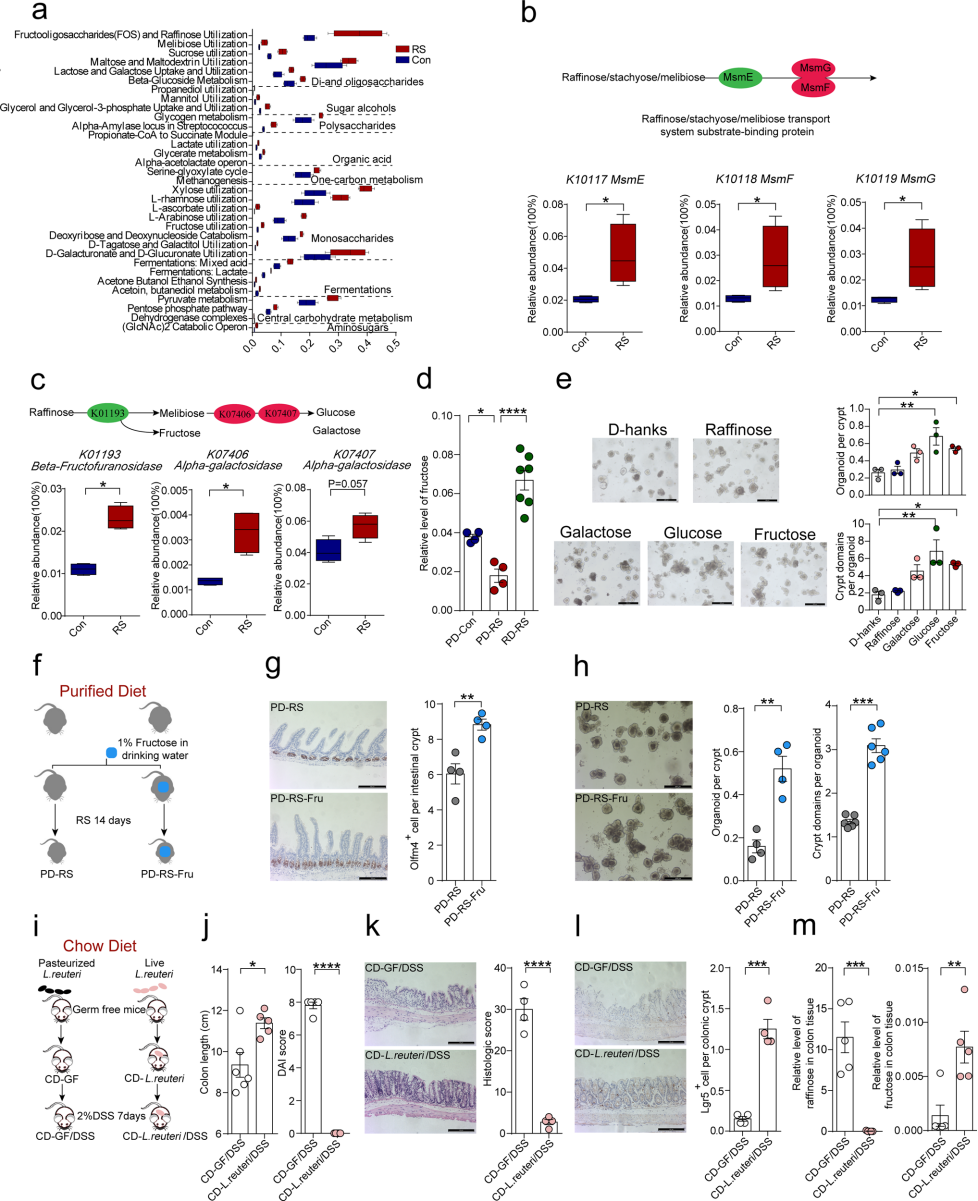
Fructose produced from raffinose promotes ISC proliferation. **a** Summary of significantly changed pathways involved in carbohydrate metabolism based on metagenomic data of fecal samples from control and stressed mice fed with chow diet. Pathways with *p* ≤ 0.05 by Wilcoxon rank-sum test are reported. **b**, **c** Box plot showing differential enrichment of indicated KOs involved in raffinose transport (**b**) and metabolism (**c**) by KEGG analysis. Box limits are the 10th and 90th percentiles, center lines are median, and the whiskers are the minimal and maximal values. Statistical analysis was performed by Wilcoxon rank-sum test for *n* = 4, *p < 0.05. **d** Relative level of fructose in the ileum of mice fed with PD or RD. Data are represented as mean ± SEM for the PD group (*n* = 4) and RD group (*n* = 7). **e** Representative images of intestinal organoids treated with blank culture (d-hanks), raffinose (1 mM), galactose (1 mM), glucose (1 mM), or fructose (1 mM). Scale bar: 200 µm. Organoids per crypt and crypt domains per crypt were represented as mean ± SEM for *n* = 3. **f** Schematic of the experimental design to explore the effect of fructose supplementation on ISC renewal. **g** Representative Olfm4 staining images of intestinal crypts and quantitation of Olfm4-positive cells per crypt. Scale bar: 100 µm. Data are represented as an average number of positive staining per crypt ± SEM for *n* = 4 (*p* = 0.0051). **h** Organoids derived from the intestinal crypts of PD-fed mice with or without fructose supplementation during stress. Scale bar: 200 µm. Data are expressed as Mean ± SEM for organoid per crypt (*n* = 4, *p* = 0.0015) or crypt domain per organoid (*n* = 6), respectively. **i** Schematic of the experimental design to understand the effect of *L. reuteri* on raffinose metabolism. **j** Disease activity index (DAI) score and colon length of mice with colitis. Data are represented as mean ± SEM for CD-GF/DSS group (*n* = 6) and CD-*L. reuteri/*DSS group (*n* = 5). *p* = 0.017 for colon length; *p* < 0.0001 for DAI. **k** Representative H&E staining of distal colon section of GF and GF-*L. reuteri* mice with colitis. Scale bar: 100 µm. Data are represented as mean ± SEM for *n* = 4 biologically independent samples. **l** Representative Lgr5 staining images and quantification of Lgr5-positive cells in the crypt of colonic tissue. Scale bar: 100 µm. Data are represented as mean ± SEM for *n* = 4 biologically independent samples. **m** Relative raffinose and fructose level in the colonic tissue of GF and *L. reuteri* colonized mice. Data are represented as mean ± SEM for *n* = 5 (*p* = 0.0003 for raffinose; *p* = 0.0071 for fructose). Statistical significance was determined by one-way ANOVA followed by Tukey’s post hoc test (**d**, **e**) or unpaired two-tailed Student’s *t*-test (**g**, **h**, **j–m**). ns no significance. *p < 0.05; **p < 0.01; ***p < 0.001; ****p < 0.0001.

The gut microbiota has evolved sophisticated metabolic capacity of diverse dietary chemicals as an adaptive response to environmental stimuli^23^. Our metagenomic data indicated that the genome of gut microbiota in CD-fed mice after stress was highly represented by pathways in carbohydrate metabolism, reflective of an increased capacity of dietary fiber disposition (Supplementary Fig. 5d). Further analysis revealed a striking change in fructo-oligosaccharide (FOS) and raffinose utilization that was intensified after stress (Fig. 5a). Raffinose is actively transported by a bacterial *Msm* transport system and hydrolyzed to d-fructose and melibiose (which sequentially degrades into d-galactose and d-glucose) by glycosidase^22^. We observed that nearly all the key genes involved in raffinose transport (*K10117*, *K10118*, and *K10119*) and hydrolysis (*K01193*, *K07406*, and *K07407*) were significantly enriched (Fig. 5b, c and Supplementary Fig. 5e), which strengthens the predicted augment in dietary raffinose utilization after stress. We also confirmed that, when PD was supplemented with raffinose, a significant increase of  intestinal fructose in stressed mice could be observed, while glucose appeared not to change and galactose was negligible (Fig. 5d and Supplementary Fig. 5f). Indeed, we further observed that in vitro incubation of raffinose (10 mM) with *L. reuteri* DSM 20016 readily produced fructose (Supplementary Fig. 5g), which suggests a reciprocal relationship between *Lactobacillus* expansion and dietary raffinose utilization in the stressed gut.

To clarify whether raffinose or its metabolic products may function to sustain the function of ISC, we exposed isolated intestinal crypts to raffinose or the three mono-saccharides, respectively. Of interest, we found that glucose and fructose (1 mM) exerted a notable effect to drive the growth and budding of cultured organoids, while raffinose and galactose showed no visible effects (Fig. 5e). Given the specific increase of fructose after raffinose supplementation to mice, we further explored the effects of fructose on intestinal epithelial renewal in stressed mice. Mice were fed on PD and fructose (1%) was provided via the drinking water (Fig. 5f). Fructose significantly increased Olfm4-positive ISC in stressed mice (Fig. 5g), and, as expected, potentiated the self-expansion of isolated intestinal crypts into organoids (Fig. 5h). Overall, these findings reveal that bacterial metabolism of raffinose to fructose may have a homeostatic role in adapting ISC function to stressful insults.

To directly address the regulatory role of *Lactobacillus*–raffinose–fructose axis on epithelial repair, we inoculated pasteurized or live *L. reuteri* into GF mice and placed the gnotobiotic mice under CD before the exposure to DSS (Fig. 5i and Supplementary Fig. 5h). Mice mono-colonized with live *L. reuteri* (CD-*L. reuteri*/DSS) showed more resilience to chemical injury to the gut, as shown by colon length and histopathological analysis (Fig. 5j, k). Notably, this benefit was associated with the preservation of stem cells in the colon of gnotobiotic mice (Fig. 5i), as well as an increased proportion of goblet cells (Supplementary Fig. 5i). We also observed that *L. reuteri* colonization led to a remarkable reduction of raffinose in the gut, accompanied by increased fructose level in the colonic tissue (Fig. 5m and Supplementary Fig. 5j), indicative of a functional metabolic axis in gnotobiotic mice.

### Fructose engages glycolysis and fuels ISC proliferation

**Fig. 6:**
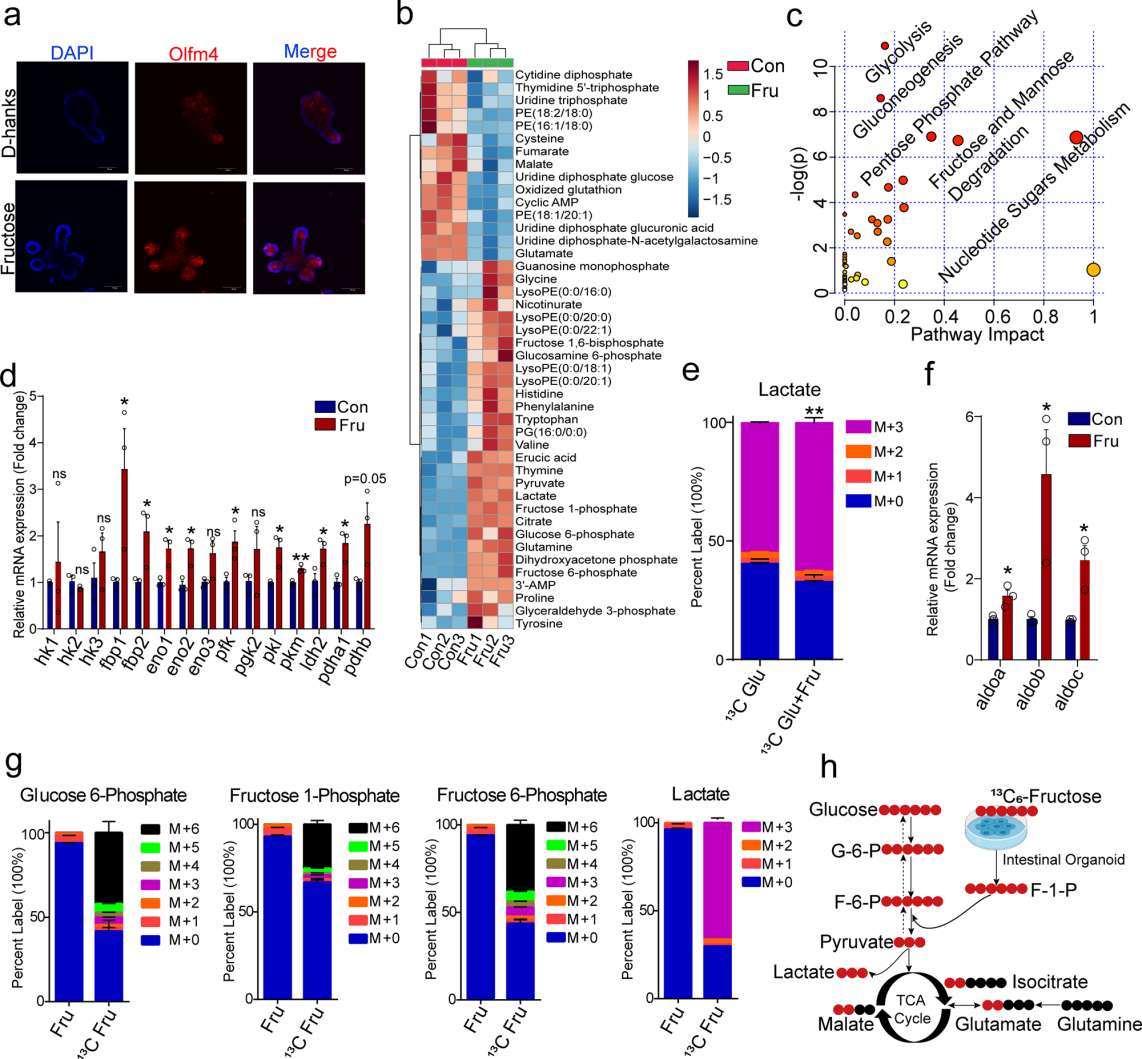
Fructose metabolism drives ISC proliferation by engaging glycolysis. **a** Representative immunofluorescent images of Olfm4 (Red) in intestinal organoids treated with or without fructose. Nuclei are stained blue. Scale bar, 50 μm. Images are representative of *n* = 3 biologically independent samples. **b** Heatmap showing a differential metabolic profile of fructose (10 mM)-treated intestinal organoids for 24 h. **c** Pathway enrichment analysis of differential metabolites from intestinal organoids treated with fructose (10 mM) as compared to PBS-treated controls by using Ingenuity Pathway Analysis (IPA). Red circles denote pathways that have significant changes. **d** Relative mRNA expression of key enzymes involved in glycolysis. *hk*, hexokinase; *fbp*, fructose-bisphosphatase; *eno*, enolase; *pfk*, phosphofructokinase; *pgk*, phosphoglycerate kinase; *pk*, pyruvate kinase; *ldh*, lactate dehydrogenase; *pdh*: pyruvate dehydrogenase complex. Data are expressed as mean fold change ± SEM for *n* = 3, unpaired two-tailed Student’s *t*-test (*p* = 0.05 for *fbp1*; *p* = 0.04 for *fbp2*; *p* = 0.01 for *eno1*; *p* = 0.01 for *eno2*; *p* = 0.08 for *eno3*; *p* = 0.03 for *pfk*; *p* = 0.02 for *pkl*; *p* = 0.001 for *pkm*; *p* = 0.04 for *idh2*; *p* = 0.01 for *pdha1*; *p* = 0.05 for *pdhb*). **e** Percent labeling of lactate following treatment of intestinal organoids with 10 mM U-[^13^C]-Glucose (^13^C Glu) after 24 h treatment with 10 mM fructose (Fru). The # in the (M + #) designation indicated how many [^12^C] were replaced with [^13^C] in the isotopic labeled metabolites. The abundance is expressed as percent labeling among all isotopomers of the metabolites. Data are expressed as mean ± SEM for *n* = 3, unpaired two-tailed Student’s *t*-test, **p < 0.01 for (M + 3) lactate. **f** Relative mRNA expression of aldonase isoforms involved in fructose utilization. *aldo*, aldolase. Data are expressed as mean fold change ± SEM for *n* = 3, unpaired two-tailed Student’s *t*-test (*p* = 0.03 for *aldoa*: *p* = 0.02 for *aldob*; *p* = 0.01 for *aldoc*). **g** Percent labeling of the indicated metabolites following treatment of mature intestinal organoids with 10 mM U-[^13^C]-Fructose (^13^C Fru) for 6 h. Data are represented as mean ± SEM for *n* = 3. **h** Schematic summary of the conversion of [^13^C]-fructose into key metabolites in glycolysis, tricarboxylic acid (TCA) cycle, and glutamine metabolism pathway as found in intestinal organoids.

Nutritional metabolites from the gut microbiota are emerging as key regulators of ISC function^24–26^. We found that fructose treatment during intestinal crypt culture effectively increased the density of Olfm4-positive stem cells in intestinal organoids (Fig. 6a). To elucidate the mechanism by which increased fructose production favors ISC proliferation, we sought to understand the metabolic reprogramming after fructose treatment, the orchestration of which proves fundamental for ISC maintenance and crypt function^24,27^. To this end, we performed untargeted metabolomics of intestinal organoids treated with fructose (10 mM) for 24 h. As expected, the metabolite profile after fructose-supplementation clustered separately from that of the control (Supplementary Fig. 6a). Typically, metabolites such as glucose-6-phosphate (G6P), fructose-6-phosphate (F6P), lactate, pyruvate, citrate, and glutamine were markedly enriched after fructose treatment (Fig. 6b). Pathway enrichment analysis revealed that the glycolysis, gluconeogenesis, pentose phosphate pathway, and nucleotide metabolism were the top four enhanced pathways induced by fructose (Fig. 6c), which may provide building blocks and signal molecules essential for cell proliferation^27^. In line with these findings, we also observed a significant increase in the mRNA expression of multiple enzymes involved in the production of glycolytic intermediates such as fructose-bisphosphatase (*fbp*), phosphofructokinase (*pfk*), and lactate dehydrogenase 2 (*ldh2*) (Fig. 6d). To gain more functional insights, we further conducted isotope tracing analysis of U-[^13^C]-glucose in intestinal organoids after exposure to fructose for 24 h. [^13^C] tracing revealed that fructose-treated organoids had a higher percentage of fully labeled lactate (M + 3), supporting the enhancement of glycolysis after fructose treatment (Fig. 6e). Together, these results indicate that fructose induces a metabolic reprogramming in intestinal organoids that favors glycolysis.

In our metabolomic assay, we also noted the presence of several metabolites previously known to be generated in the hepatic disposition of fructose, namely fructose-1-phosphate (F1P) and dihydroxyacetone phosphate (DHAP) (Fig. 6b and Supplementary Fig. 6b). Although the fructose metabolism pathway is well characterized in the liver and some tumors, its presence and role in intestinal organoids are mostly unknown^28,29^. We confirmed the presence of aldolase B in intestinal organoids, the mRNA expression of which was induced by more than four folds after fructose treatment (Fig. 6f). We, therefore, reasoned that the higher glycolytic rate from [^13^C]-Glu in intestinal organoids treated with fructose could be attributed to its transforming to glycolytic substrates. To test this, we determined the metabolic fate of fructose in intestinal organoids using ^13^C isotopic tracing. F1P was partially ^13^C-labeled at all six positions (M + 6), confirming the activity and presence of fructokinase in intestinal organoids (Fig. 6g). ^13^C-incorporation was also identified in multiple glycolytic intermediates (G6P, F6P, pyruvate) as well as the terminal product lactate (Fig. 6g and Supplementary Fig. 6c), supporting the direct entry of fructose into the glycolytic pool in intestinal organoids. Label incorporation of (M + 2) isocitrate, fumarate, and malate were also observed (Supplementary Fig. 6c), indicating that fructose also contributes directly to key metabolites in the TCA cycle. Glutamate and aspartate also displayed enrichment from U-[^13^C] fructose, suggesting that fructose may provide an additional source for amino acid synthesis (Supplementary Fig. 6c). These findings indicate that fructose engages glycolysis and transforms into the intermediates involved in central carbon metabolism in intestinal organoids (Fig. 6h), which may sustain ISC proliferation.

## Discussion

Nutritional and social factors are believed as major drivers of an increased prevalence of gut disorders in modern society. How diets interact with psychological stressors to affect gut pathophysiology and the causal dynamics are poorly understood. Our study provides mechanistic insights that dietary components and chronic stress interactively modulate gut microbial metabolism and its crosstalk with ISCs. In particular, we identify that dietary raffinose and *L. reuteri* constitute a metabolic feedforward circuit promoting ISC proliferation via fructose-augmented and engaged glycolysis. Our data, therefore, shed light on the dynamic nature of psychological stress-gut microbe crosstalk in adaption to host diets, which highlights the importance of diet-microbe interplay in dictating gut response to psychological stress.

Gut homeostasis is exceptionally prone to the perturbation by psychological stressors^5^. Previous studies have shown altered gut microbiota in response to stressful events, but the modifying effects of co-existing factors are largely underappreciated^30,31^. We find that PD, but not CD, critically drives the deleterious impact of chronic stress on ISCs renewal and gut homeostasis. It is, therefore, reasonable to suggest that researches into the intricate link between stress and gut disorders should consider specific experimental conditions, especially diet, that may confound the change of gut microbiota. By transplanting the fecal material of CD-fed mice to GF recipients, we confirmed a direct role of stressed-shaped gut microbiota in modulating epithelial response to injury. Notably, we observed a consistent enrichment of *Lactobacillus* spp., which are fostered by CD but not PD, accompany with attenuated colon epithelial injury in specific pathogen-free (SPF) and GF models. These findings suggest that strengthening the adaptive enrichment of *Lactobacillus*, possibly via dietary adjustment, may increase the individual resilience to psychological stress-induced gut disorders.

The dietary composition is likely one of the most common and powerful environmental factors affecting gut commensals and human health^8^. Supporting this view, a recent study of 32 custom diets in mice showed that changes in dietary protein and fiber are major determinants of disease outcome in the DSS colitis model^15^. Here we uncover that raffinose from CD sustains the growth of *Lactobacillus* species under psychological stress and upon DSS-induced chemical injury. Given the chemical complexity of diets with a large fraction of nutritional components remaining unmapped, it is highly possible that other dietary components such as polyphenols are involved in shaping microbial dynamics during stress, most likely in concert with raffinose. The gut microbiota in turn has evolved a unique metabolic capability of nutrients which exerts profound effects on host physiology^32^. Our findings support a scenario in which host diet, via constituting a feedforward metabolic circuit with the *Lactobacillus*, has a major role in determining gut epithelial vulnerability to psychological stress. This mechanistic link expands the accumulating appreciation of diet-microbe interplay under stressful conditions^7,8^, further explaining why dietary pattern finely tunes the prevalence of functional gut disorders and mental diseases in modern society^33^.

Efficient nutrient acquisition and utilization are essential for microbial persistence in the gut and adaption to environmental changes^34^. In line with this fact, our metagenomic assay suggested that the gut microbiota from CD-fed mice showed an enhanced capacity of oligosaccharide utilization after chronic stress, possibly reflective of an adaptive demand for energy processing. It would be of interest to understand how *Lactobacillus* spp. integrate the cues from diet and stress to mount a dynamic change in abundance and nutrition utilization. One intriguing possibility is that the metabolism of dietary raffinose may confer colonization fitness of *Lactobacillus* in the stressed gut. Indeed, microbes rely on their community to sustain essential biologic activities^35^. It is, therefore, envisioned that a network of microbes across different phyla is involved in coordinating the bloom of *Lactobacillus* strains and the disposition of dietary components in the stressed gut, which deserves more insights in future studies.

Probiotics supplementation represents a major route of gut microbiota-based therapies, although several challenges remain in this frontier^10^. Our findings in GF mice colonized with a live *Lactobacillus* strain highlight that CD is useful to foster the function of *Lactobacillus* spp. to maintain a health-promoting diet-microbe interplay. A recent study also shows that a 4-day fasting-mimicking diet stimulated protective gut microbiota represented by *Lactobacillus* strains^36^. Of interest, a previous study also unveils resistant starch as a dietary inhibitor of *Lactobacillus* abundance^37^. These facts reinforce the importance of proper dietary design to support the colonization and activity of gut commensals, with the aim to optimize the benefit of probiotic-based therapy^38^. In this light, raffinose, as a prebiotic for stimulating the growth of *Lactobacilli*, could become a promising food supplement for irritable bowel syndrome patients and other stress-liable populations.

To maintain gut epithelial homeostasis, the ISCs constantly undergo self-renewal and differentiation in a highly regulated manner^39^. Our findings coincide with a paramount role of gut microbial metabolites of nutrition in shaping ISC function and add fructose to this list of signaling metabolites. Specifically, our study shows that fructose derived from dietary raffinose favors ISC proliferation in cultured intestinal organoids as well as the crypts from the stressed gut, which is partially linked to its augmenting and engaging glycolysis to provide fuel for stem cell renewal. As previously reported, glycolysis in Paneth cells provides lactate for enhanced mitochondrial oxidative phosphorylation in stem cells to support their optimal function^27^. In this light, additional work is needed to fully dissect how fructose metabolism is integrated into the metabolic network for proper ISC functioning, especially in the crypt niche where cellular interaction occurs between Paneth cells/stromal cells and stem cells. Moreover, how different cells differentially exploit fructose-augmented/engaged glycolysis to drive the crypt growth is another intriguing issue. A complex signaling network exists to orchestrate the expansion of ISCs at the crypt and their differentiation toward secretory lineages including goblet cells and Paneth cells^40^. It would therefore be of interest to explore whether fructose may activate canonical signals (e.g., Wnt, mTOR) to orchestrate energetic demand and regulate ISC proliferation and differentiation under stressful conditions.

In our study, raffinose supplementation in PD successfully increased fructose level in the intestine. Of interest, a previous study showed that a large majority of dietary fructose is metabolized by the intestine before reaching the circulation^41^. Therefore, oral supplementation of raffinose appears to be optimal to deliver fructose at sufficient quantity. Excessive consumption of a fructose-enriched diet carries the risk of causing metabolic disorders such as nonalcoholic fatty liver disease and increases the risk of tumorigenesis^29^. Our study, however, indicates that fructose derived from dietary oligosaccharides such as raffinose can be beneficial for sustaining ISC during stressful life events. Indeed, it was recently uncovered that a certain proportion of dietary fructose is converted by the gut microbiota to acetate^42^, which raises the possibility of fructose feeding of the microbes. We, therefore, suggest that dietary supplementation of raffinose and/or fructose at an appropriate dose can exert beneficial effects to the stressed gut, and future studies will be necessary to assess whether this could translate to viable dietary/nutraceutical approaches to sustain the renewal of intestinal epithelia that are vulnerable to various environmental and psychological insults.

There is growing interest in understanding the interconnected regulation of gut microbiota by co-existing host inputs. Our work reveals a feed-forward relationship between raffinose metabolism and *Lactobacillus* strains that causally links diet, psychological stress, and ISC maintenance. These findings emphasize the need to take a dietary perspective in manipulating the health risk of psychological stress in modern society. Additional experiments carried out in humans are also anticipated to validate the therapeutic value of such a diet-microbial metabolism feedforward loop in gut disorders.

## Methods

### Mice and diets

Six-week-old female SPF Balb/c mice were obtained from Vital River Laboratory Animal Co., Ltd (Beijing, China). On arrival, all the mice were randomly housed in individually ventilated-plastic cages (5 to 6 mice per cage) in the Animal Facility of China Pharmaceutical University, with 12/12 h light/dark cycle, controlled temperature at 20–22 °C, 45 ± 5% humidity and free access to water and food. Mice at 7~8 weeks of age were used for the experiments. Germ-free Balb/c mice were bred in flexible-film plastic isolators at the Shanghai SLAC Laboratory Animal Co., Ltd (Shanghai, China), provided with a regular 12 h dark/light cycle (lights on at 06:00 AM) and sterile normal chow diet ad libitum. In fecal microbiota transplant experiments, female mice at 7 to 9 weeks of age were used as recipients for the fecal materials of female SPF mice. For *Lactobacillus* colonization, both male and female mice were used and randomly assigned to different experimental groups. All the animal experiments were approved by the Institutional Animal Care and Use Committee of China Pharmaceutical University.

Three diets were used during the experiment: rodent chow diet (CD), composed of 9% fat, 22% protein, and 69% carbohydrate, was purchased from Jiangsu Xietong Pharmaceutical Bio-engineering Co., Ltd. (Nanjing, China); Isocaloric purified diet (PD, AIN-93G), composed of 10% fat, 20% protein, and 70% carbohydrate, was purchased from Nantong Trophic Animal Feed Co., Ltd (Nantong, China); Purified diet (AIN-93G) supplemented with raffinose (100 g/kg, Amresco)^43^, was prepared and provided by Nantong Trophic Animal Feed Co., Ltd. The mice were maintained on a separate diet for at least 1 week before the initiation of the experiment.

### Bacterial strains

*Lactobacillus reuteri* for colonization to germ-free mice or PD-fed mice was isolated from the feces of CD-fed mice. *Lactobacillus* strains were firstly isolated with De Man, Rogosa, and Sharpe (MRS, Hope Bio-tech, cat# HB0384-5) selective media, under anaerobic conditions for solid media and 5% CO_2_ (85% N_2_, 10% H_2_) for liquid media at 37 °C. After further selective growth in LAMVAB agar (Hope Bio-tech, cat# HB8744) and Brain Heart Infusion (BHI) agar, single colonies were isolated and sequenced. The strain used was identified as *Lactobacillus reuteri* DSM 20016 by sequencing (details described in Supplementary Fig. S4d). For colonization, *Lactobacillus reuteri* DSM 20016 was cultured in sterile MRS broth at 37 °C for 24 h in the anaerobic chamber. Cultures were washed with anaerobic PBS and concentrated to 10^9^ CFU/mL with fresh MRS broth under anaerobic conditions.

### Induction of chronic stress and colitis in mice

Chronic restraint stress (RS) in mice was performed as previously reported^44,45^. Briefly, mice were placed inside a plastic mouse holder for 4 h daily, from 10:00 am to 14:00 pm, and repeated for a total of 14 consecutive days. During non-stress periods, all mice were conventionally housed and allowed free access to food and water. To induce epithelial injury and colitis, mice were subsequently exposed to 2% (wt/vol) DSS (36,000–50,000 Da, MP Biomedicals) in drinking water for 7 days, followed by pure drinking water for 3 days. Body weight, stool consistency, rectal bleeding, and general appearance of mice were monitored. Disease activity index (DAI) was assessed on the day of sacrifice based on the overall changes of body weight, rectal bleeding, and fecal morphology, as previously described^5^.

### Gut microbiota profiling by 16S rRNA sequencing

Bacterial DNA from fecal samples was extracted using the PowerSoil DNA Isolation kit (Qiagen) and quantified for concentration adjustment. PCR was performed on the aliquoted DNA using the V4 region of the 16S rRNA gene (515 F and 806R). A standard thermocycler protocol was used: 95 °C for 2 min, followed by 25 cycles of 1 min at 95 °C, 1 min at 55 °C, and 1 min 72 °C, with a final 5 min at 72 °C and hold at 4 °C. Amplifications were purified using the QIAquick PCR purification kit (Qiagen). Purified 16S rRNA amplicons were pooled in equimolar amounts, and the amplicon size was determined by an Agilent 2200 bioanalyzer. Pools were sequenced with a 600 cycle kit on the Illumina MiSeq (Illumina Inc.). Raw fastq files were demultiplexed and quality-filtered using QIIME (Quantitative Insights into Microbial Ecology, version 4.2; http://drive5.com/uchime/uchime_download.html) as previously described, and the sequences were picked against a high-quality 16S rRNA sequence from the Green Genes database after trimming the primer, barcode, and chimeras. Operational taxonomic units (OTUs) were picked at 97% similarity cutoff using UPARSE (version 7.1; http://drive5.com/uparse/), and chimeric sequences were identified and removed using UCHIME. Alpha diversity was estimated on the basis of the gene profile of each sample according to the Shannon index; while Beta-diversity was estimated by calculating Bray–Curtis dissimilarity between samples. Total sum scaling was used for data normalization and two variables were used in PCoA plotting of bacterial OTU in fecal samples.

### Metagenomics

Total genomic DNA was extracted from fecal samples using the E.Z.N.A.^®^ Soil DNA Kit (Omega Bio-tek) according to the manufacturer’s instructions. The concentration and purity of extracted DNA were determined with TBS-380 and NanoDrop2000, respectively. DNA extract quality was checked on 1% agarose gel. DNA extract was fragmented to an average size of about 300 bp using Covaris M220 (Gene Company Limited, China) for paired-end library construction. A Paired-end library was constructed using NEXTFLEX Rapid DNA-Seq (Bioo Scientific). Adapters containing the full complement of sequencing primer hybridization sites were ligated to the blunt-end of fragments. Paired-end sequencing was performed on Illumina NovaSeq/Hiseq Xten (Illumina) at Majorbio Bio-Pharm Technology Co., Ltd. (Shanghai, China). Sequence data associated with this project have been deposited in the NCBI Sequence Read Archive (SRA) repository.

The original sequencing data were evaluated by FastQC, and Trimmomatic filtering was evaluated to obtain relatively accurate and effective data. Open reading frames (ORFs) from each assembled contig were predicted using Prodigal. The predicted ORFs with the length being or over 100 bp were retrieved and translated into amino acid sequences using the NCBI translation table. Representative sequences of non-redundant gene catalog were aligned to NCBI NR database with *e*-value cutoff of 1 e^−5^ using BLASTP (Version 2.2.28^+^) for taxonomic annotations. Functional profiles were generated with the SEED subsystems database with an e-value cutoff of 1 e^−5^. The KEGG annotation was conducted using BLASTP (Version 2.2.28^+^) against the Kyoto Encyclopedia of Genes and Genomes database (http://www.genome.jp/keeg/) with an *e*-value cutoff of 1 e^−5^.

### Histological, immunohistochemical, and cytokine analysis

After sacrifice, the distal colon and ileum of mice were harvested, fixed in 4% polyformaldehyde, and processed for histological and immunohistochemical analysis. H&E stained intestinal and colonic sections were routinely assessed for the length of villus size and crypt density; Alcian blue-stained sections were observed for the number of goblet cells. The change of stem cells and Paneth cells density were observed by immunohistochemical analysis of Olfm4 (intestinal tissue)/Lgr5 (colonic tissue) and Lysozyme, respectively. Briefly, sections were deparaffinized by heating at 60 °C for 30 min, and rehydrated in a graded ethanol series of decreasing ethanol concentrations. Sections were then subjected to heat-induced epitope retrieval (10 mM sodium citrate buffer-0.05% Tween 20, pH 6.0), blocked, and incubated with the primary antibodies overnight at 4 °C. The primary antibodies used were rabbit anti-Olfm4 (1:500; CST)/anti-Lgr5 (1:200; Abcam) and rabbit anti-Lysozyme antibody (1:150; Abcam). Images were captured using a Leica DMI 3000B light microscope (Leica, Germany) in a blinded manner. Histopathological scores were determined in the colon section as previously described^46^. Crypt and villus length were measured from the bottom of the crypt to the crypt-villus junction and from the crypt-villus junction to the tip of the villus with Image J software (NIH). Quantification of goblet cells, lysozyme, Lgr5, and Olfm4-positive cells were counted from ~40 intact, well-orientated crypts per mouse. For cytokine determination in the colon, tissue homogenates were made in ice-cold PBS with protease inhibitor and centrifuged at 12,000×*g* at 4 °C for 15 min. The concentration of IL-1β, IL-6, TNF-α, and MCP-1 were measured using commercially-available ELISA Kits (ExCell Bio, Shanghai, China) following the manufacturer’s instructions.

### Fecal microbiota transplant to germ-free mice

Immediately following the final cycle of restraint stress on the 14th day, conventional mice were euthanized via CO_2_ asphyxiation. An equivalent amount of cecal contents (0.5 g) from control and stressed mice was pooled in 15 mL of anaerobically pre-reduced sterile Ringer working buffer (9 g/L of sodium chloride, 0.4 g/L of potassium chloride, 0.25 g/L of calcium chloride dehydrate, and 0.05% (w/v) l-cysteine hydrochloride) in an anaerobic chamber (85% N_2_:5% CO_2_:10% H_2_). The cecal material was suspended by thorough vortex (5 min) and settled by gravity for 5 min. The clarified supernatant was transferred to a clean tube, and an equal volume of 20% (w/v) skimmed milk (LP0031, Oxoid, UK) was added. The inoculum was freshly prepared on the day of an experiment, with the rest stored at −80 °C until the second inoculation. Female germ-free (GF) Balb/c mice were maintained on a sterilized normal chow diet and were randomly assigned into two isolators for acclimatization. After 1 week, mice were orally gavaged with 200 μL of cecal contents from stress-exposed donors or non-stressed control donors (*n* = 6).

### *Lactobacillus reuteri* inoculation in germ-free mice

For *Lactobacillus reuteri* inoculation, GF Balb/c mice received 200 μL of *Lactobacillus reuteri* DSM 20016 suspension (10^9^ CFU/mL in PBS). Inoculation was repeated on the next day to reinforce the microbiota colonization. After 1 week, the feces were freshly collected and cultured using the spread plate method on BHI agar at 37 °C under aerobic or anaerobic conditions for 48 h. The mice were then given sterile drinking water supplemented with 2.5% (wt/vol) sterile DSS for 7 days, followed by sterile drinking water for 3 days.

### In vitro growth assay of *Lactobacillus reuteri*

*Lactobacillus reuteri* DSM 20016 was cultured in a sterile MRS plate in a 37 °C anaerobic incubator for 2 days. A single colony was used to inoculate 5 mL BHI media, and seed cultures were shaken overnight at 37 °C. *Lactobacillus reuteri* was cultured in anaerobic tubes in the presence or absence of Raffinose (100 µM and 1 mM) and OD 600 value was measured every 2 h.

### Isolation and culture of mouse intestinal organoids

Intestinal crypts were isolated as described previously^47^. Briefly, after sacrificing mice, 10 cm of small intestine proximal to the stomach were cut and washed in ice-cold d-PBS (HyClone). Crypts and villi were exposed by cutting the intestines into small 2 mm pieces, followed by extensive washes (15–18 times) to remove contaminants. The pieces were then resuspended in Gentle Cell Dissociation Reagent (Stemcell technologies) and incubated at room temperature for 15 min to release most of the crypts. Next, the crypts were gently filtered through a 70 µm filter (Falcon, Cat #352350), and isolated crypts were collected in the crypt culture medium, counted, embedded in matrigel (GFR and Phenol Red-Free Basement Membrane Matrix, Corning), and cultured in IntestiCult organoid growth medium (Stemcell Technologies). The number of intestinal organoids was observed at days 2, 5, and 7 of culture. To investigate the effect of dietary or microbial components, organoids were incubated with raffinose, glucose, fructose, galactose (1 mM) in organoid growth medium (mixed with organoid growth medium at a 1:50 ratio), respectively. Immunofluorescence imaging of Olfm4 in intestinal organoids was performed following the procedure previously described by our group^48^.

### Metabolomics study of intestinal organoids

On the 5th day of secondary organoid culture, the organoids were treated with fructose (10 mM d-fructose)-containing IntestiCult organoid growth medium for 24 h and washed with pre-cooled PBS before harvesting. Sample preparation and UPLC-Q-TOF/MS (Synapt G2si, Waters) based metabolomic study of the intestinal organoids were performed following the method previously described by our group and others^49,50^. LC separation was achieved on an XBridge BEH Amide column (4.6 mm × 100 mm, 3.5 μm; Waters) using a gradient of solve A (10 mM ammonium acetate and10 mM ammonium hydroxide in 95:5 water/acetonitrile mixture, pH = 9) and solvent B (acetonitrile). The flow rate was 0.4 mL/min. The gradient was: 0 min, 85% B; 2 min, 85% B; 8 min, 30% B; 11 min, 30% B; 14 min, 85% B; 20 min, 85% B. The mass spectrometer was operated with the spray voltage of −2.8 kV in negative mode. Cone gas and desolvation gas were set at 500 and 800 L/h, respectively. The source temperature was 120 °C. Fast data-dependent acquisition (DDA) MS/MS experiments were performed with collision energy map which included low mass ramp start 5 eV from 10 eV and high mass ramp start 40 eV from 65 eV CE. The Progenesis QI (Nonlinear Dynamics, Newcastle, UK) was used for peak picking and alignment to screen the metabolic biomarkers that displayed significant changes between the control and the fructose-treated group. Molecular identification of the assigned biomarkers was accomplished by matching the acquired precursors and fragment ions against several standard metabolome database including the Human Metabolome Database (http://www.hmdb.ca/), MassBank (http://www.massbank.jp/index.html), and METLIN (http://metlin.scripps.edu/index.php). Partial metabolite identification was further confirmed by comparison with available standards. Metabolic pathway enrichment analysis of these identified metabolic biomarkers was carried out by MetaboAnalyst 3.0. (http://www.metaboanalyst.ca/faces/ModuleView.xhtml).

### Isotope tracing analysis in intestinal organoids

For metabolic flux analysis of ^13^C-labeled glucose, intestinal organoids were pretreated with unlabeled fructose (10 mM) for 24 h, washed with PBS, and then switched into ^13^C-labeled glucose-containing DMEM/F12 medium (10 mM U-[^13^C] d-glucose, Cambridge Isotope Laboratories) for 6 h. To trace the metabolism of fructose, intestinal organoids were cultured in unlabeled fructose-containing DMEM/F12 medium (10 mM d-fructose, 0 mM d-glucose, 2.5 mM l-glutamine) for 12 h, and then switched into ^13^C-labeled fructose-containing medium (10 mM U-[^13^C] d-fructose, Sigma-Aldrich) for 6 h. To harvest intracellular metabolites, organoid samples were washed with pre-cooled PBS three times and the metabolism was quenched with 80% ice-cold methanol. Pre-cooled methanol (1 mL) with para-chlorophenylalanine (1 µM) as the internal standard was added to each sample and incubated on ice for 10 min. Sample lysates were then centrifuged at 4 °C for 15 min at 23,000×*g*. The supernatants were removed and evaporated under vacuum before LC–MS analysis. The UPLC conditions for isotope tracing analysis were the same for the metabolomic study of the intestinal organoids as described above. For mass spectrometry detection of isotopic ions, only the parent ions were monitored without multiple fragmentations, and MS source parameters were set as follows: cone gas 500 L/h, ion source temperature 120 °C, desolvation gas 800 L/h, spray voltage −2.8 kV, mass to charge (*m*/*z*) range 50–1200.

### Dietary composition analysis

Diet pellets (30 mg) were weighted by adding 400 µL of methanol (pre-cooled at −20 °C), vortexed for 60 s, and then centrifuged for 10 min at 13,000×*g*, 4 °C. The supernatant was evaporated to dryness under vacuum, reconstituted with 150 μL of methanol aqueous solution containing 2-chlorophenyalanine (4 ppm). The sample was filtered through a 0.22 μm membrane before analysis on a Thermo Q Exactive Focus mass spectrometer (LC–MS/MS). Chromatographic separation was achieved on an ACQUITY UPLC^®^ HSS T3 (150 × 2.1 mm,1.8 μm; Waters) column maintained at 40 °C. Column separation of analytes was achieved by gradient elution following the program: 2% B over 0–1 min, 2%~50% B over 1–9 min, 50%~98% B over 9–12 min, 98% B over 12–13.5 min, 98%~2% B over 13.5–14 min, and 2% B over 14–20 min in the positive mode (or over 14~17 min in the negative mode), where A denotes water with 0.1% formic acid and B denotes  acetonitrile with 0.1% formic acid for positive mode, and A denotes 5 mM ammonium formate and B denotes acetonitrile for negative mode. The flow rate was 0.25 mL/min. The mass spectrometer was operated with the spray voltage of 3.8 and −2.5 kV in positive and negative modes. The value of sheath gas and auxiliary gas were set at 45 and 15, respectively. The capillary temperature was 325 °C. The Orbitrap analyzer scanned over a mass range of *m*/*z* 81–1000 for a full scan at a mass resolution of 70,000. Data-dependent acquisition (DDA) MS/MS experiments were performed with a higher energy collision-induced disassociation (HCD) scan. The normalized collision energy was 30 eV.

### Glucose, fructose, and galactose analysis in enteric tissue

Glucose, fructose, and galactose in enteric tissue samples were detected using UPLC-Q-TOF/MS (Waters). Briefly, jejunum, ileum, or colon tissue (30 mg) was transferred to homogenizer (pre-cooled at −20 °C) and 100 µL of H_2_O (pre-cooled at 4 °C) was added for homogenization for 1 min. Next, 800 µL methanol solution containing 4-chlorophenylalanine (1 μg/mL) were added for protein precipitation and vortexed for 10 min. The sample lysates were centrifuged at 30,000×*g* for 10 min at 4 °C and dried under vacuum. It was reconstituted with 100 μL of methanol: water solution (50:50, v/v) before analysis. An XBridge BEH Amide HPLC column (100 mm × 4.6 mm, 3.5 μm; Waters) was used. The column was maintained at 40 °C. The injection volume was 10 μL and the flow rate was 0.6 mL/min. The mobile phase A consisted of 95% of 5 mM ammonium acetate buffer, and 5% acetonitrile (pH = 9), and B was acetonitrile. The gradient was as 0–3 min, 85% B; 3–6 min, 85~30% B; 6–11 min, 30~2% B; 11–13 min, 2% B; 13–14 min, 2~85% B; and 14–21 min, 85% B. The glucose, fructose, and galactose in samples were detected using QTOF-MS (Xevo G2-XS, Waters) operating in negative ion mode. Raw data for glucose, fructose, and galactose quantification were obtained with MassLynx v4.1 and analyzed by Quanlynx v4.1(Waters, Milford, MA).

### Quantitative real-time PCR

Total RNA was extracted from a longitudinal section of colon tissues with RNAiso Plus reagent (Takara, cat#9108) according to the manufacturer’s instructions. cDNA preparation, purity assessment and quantitation, and real-time PCR were performed as previously described by our group^48^. The primer sequences were shown in Supplementary Table 1. The expression of target genes was normalized to the expression of *Gapdh*, and shown as fold change relative to the control group based on the 2^−*△△*Ct^ method.

### Statistics

GraphPad Prism version 6.0 (GraphPad Software, San Diego, USA) was used for statistical analysis unless otherwise indicated. For microbiome analysis, Principal-component analysis, Random forest, LEfSe analysis were conducted using the web-based tool MicrobiomeAnalyst 4.0 (www.microbiomeanalyst.ca) to compare the microbial composition and discover microbiome biomarker. For animal studies, the sample size was estimated from previous studies and no statistical test had been used to predetermine the sample size. A two-tailed Student’s *t*-test was used to evaluate statistical significance between the two groups. For multiple group comparison, one-way analysis of variance (ANOVA) followed by Tukey’s post hoc test was performed unless otherwise indicated. Non-parametric tests were employed when analyzing the relative abundance in the genus. For the non-parametric tests, Wilcoxon rank-sum test was used to evaluate statistical significance between two groups, and the Kruskal–Wallis test was used to analyze differences among three or more experimental groups. Statistical significance is represented by *p < 0.05, **p < 0.01, ***p < 0.001, and ****p < 0.0001. Sample size and statistical tests are also indicated in the figure legends.

### Reporting summary

Further information on research design is available in the Nature Research Reporting Summary linked to this article.

## Supplementary information

Supplementary Information

Reporting Summary

## Data Availability

The 16S rRNA and metagenomic sequencing dataset are available from the NCBI SRA database with the accession numbers PRJNA675376, PRJNA675375, PRJNA675371, PRJNA675369, and PRJNA675416. Raw data for metabolomics are deposited to Metabolomics Workbench with the Project ID: PR001041. Source data are provided with this paper.

## Source data

Source Data
