# Supplementary material for: A diet-microbial metabolism feedforward loop modulates intestinal stem cell renewal in the stressed gut
Source: Nat Commun. 2021 Jan 11;12:271. doi: 10.1038/s41467-020-20673-4 (PMC7801547; doi:10.1038/s41467-020-20673-4)
Supplement: Supplementary file 1 — Supplementary Information [file 41467_2020_20673_MOESM1_ESM.pdf]

A Diet-Microbial Metabolism Feedforward Loop Modulates Intestinal Stem Cell Renewal in the Stressed Gut

Hou *et al.*,

Supplementary Figures 1-6

Supplementary Table 1

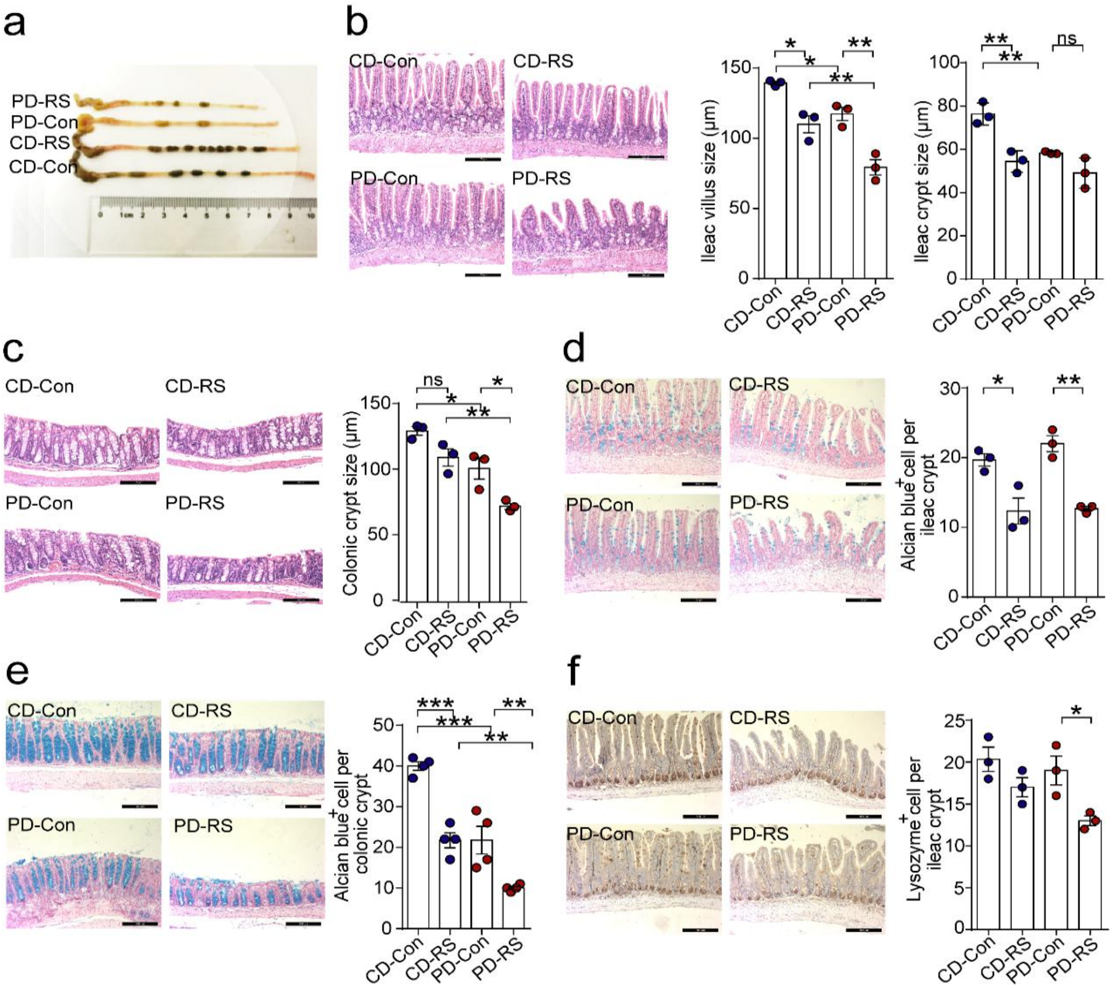

Supplementary Fig.1. Dietary pattern impacts the effect of chronic stress on crypt size and epithelial differentiation

(**a**) Representative images of colon at the end of 14 days restraint stress.

(**b, c**) H&E staining images and quantification of the villus and crypt size in the ileum (**b**) and colon (**c**). ~40 crypts per tissue sample were included for analysis (n=3). Scale bar: 100 μm.

(**d, e**) Alcian blue mucin staining images and quantification of ileac (**d**) and colonic (**e**) goblet cells per crypt (n=3). Scale bar: 100 μm. In (**d**): CD-Con *vs* CD-RS, p=0.01; PD-Con *vs* PD-RS p=0.002; In (**e**): CD-Con *vs* CD-RS, p=0.0002; CD-Con *vs* PD-Con, p=0.0002; CD-RS *vs* PD-RS, p=0.0067; PD-Con *vs* PD-RS, p=0.0067.

(**f**) Lysozyme-staining images and quantification of Paneth cells at the bottom of intestinal crypts (n=3). Scale bar: 100 μm. All the data are represented as Mean ± SEM. Statistical significance was determined by one-way ANOVA followed by Tukey's *post-hoc* test. *$p < 0.05$, **$p < 0.01$, ***$p < 0.001$, ****$p < 0.0001$.

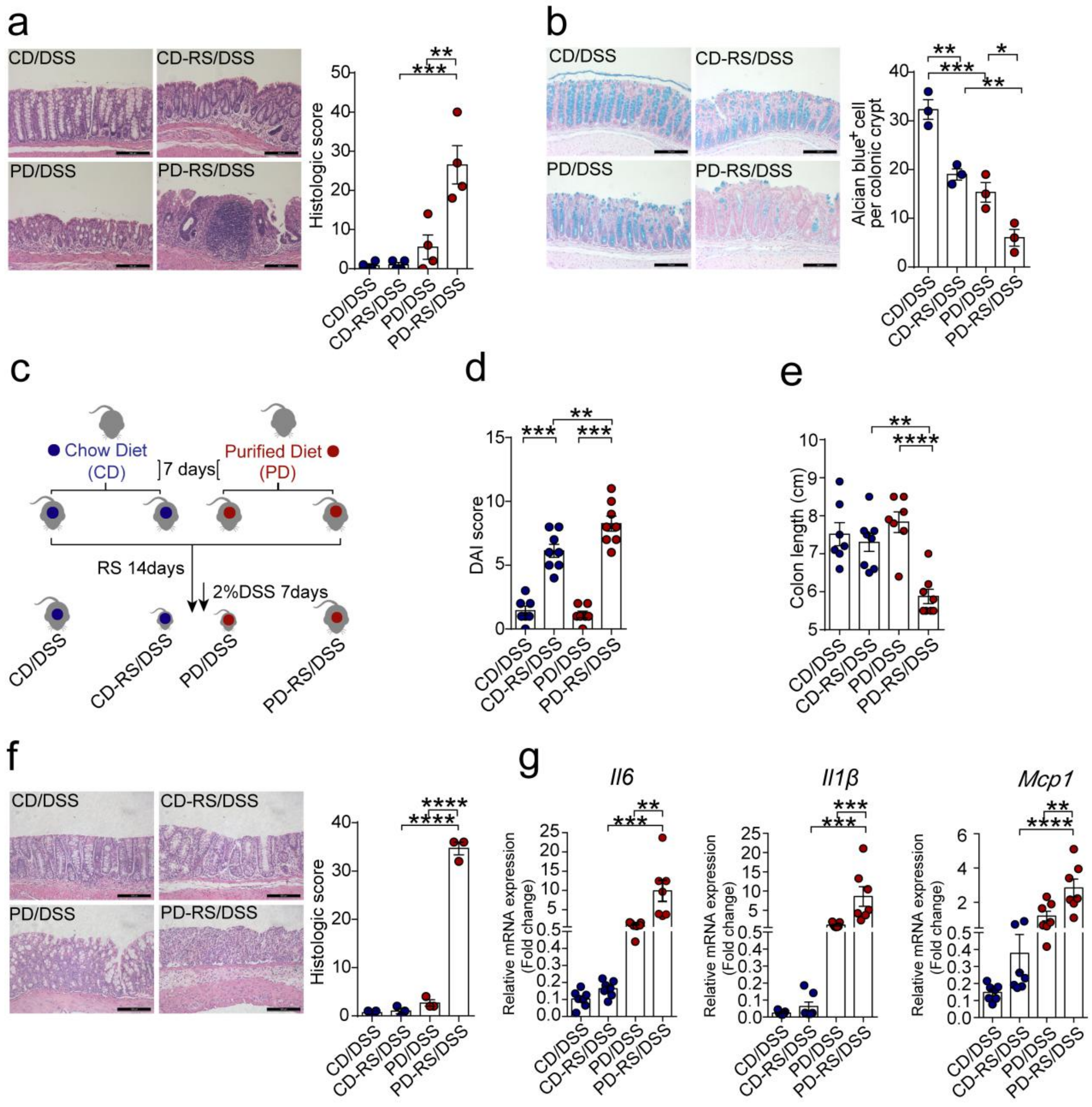

Supplementary Fig.2 Dietary pattern modulates gut epithelial susceptibility to chemical injury after or during restraint stress

(**a**) Representative H&E staining images and histologic score of distal colon section of stressed mice after exposure to DSS. Scale bar: 100 µm. Data are represented as Mean ± SEM for n=4 biologically independent samples (CD-RS/DSS *vs* PD-RS/DSS, *p*=0.0002; PD-DSS *vs* PD-RS/DSS, *p*=0.0013).

(**b**) Alcian blue (AB) mucin staining of distal colon section on day 7 of DSS exposure. Scale bar: 100 µm. Data are represented as Mean ± SEM for n=3 biologically independent samples (CD/DSS *vs* CD-RS/DSS, *p*=0.003; CD/DSS *vs* PD/DSS, *p*=0.0006; CD-RS/DSS *vs* PD-RS/DSS, *p*=0.003; PD-DSS *vs* PD-RS/DSS, *p*=0.02).

(**c**) Schematic of the experimental design. Mice were restraint stressed for 14 days, and during the last 7 days of restraint stress (RS), mice were also exposed to 2% DSS in the drinking water.

(**d**,**e**) Disease activity index (DAI) scores (**d**) and colon length (**e**) of colitis mice. Data are represented as Mean ± SEM for control group (n = 7) and stress group (n = 8). For DAI: CD/DSS *vs* CD-RS/DSS, $p<0.0001$; CD-RS/DSS *vs* PD-RS/DSS, $p=0.01$; PD-DSS *vs* PD-RS/DSS, $p<0.0001$; For colon length: CD-RS/DSS *vs* PD-RS/DSS, $p=0.0016$; PD-DSS *vs* PD-RS/DSS, $p<0.0001$.

(**f**) Representative H&E staining and histologic score of distal colon section of mice with DSS exposure during the last 7 days of restraint stress. Scale bar: 100 µm. Data are represented as Mean ± SEM for n=3 biologically independent samples.

(**g**) Relative mRNA expression of *Il6*, *Il1β* and *Mcp1* in the colon tissue of colitis mice. Data are represented as average fold change ± SEM for n=7.

Statistical significance was determined by one-way ANOVA followed by Tukey's post-hoc test. *$p < 0.05$, **$p < 0.01$, ***$p < 0.001$, ****$p < 0.0001$; ns, no significance.

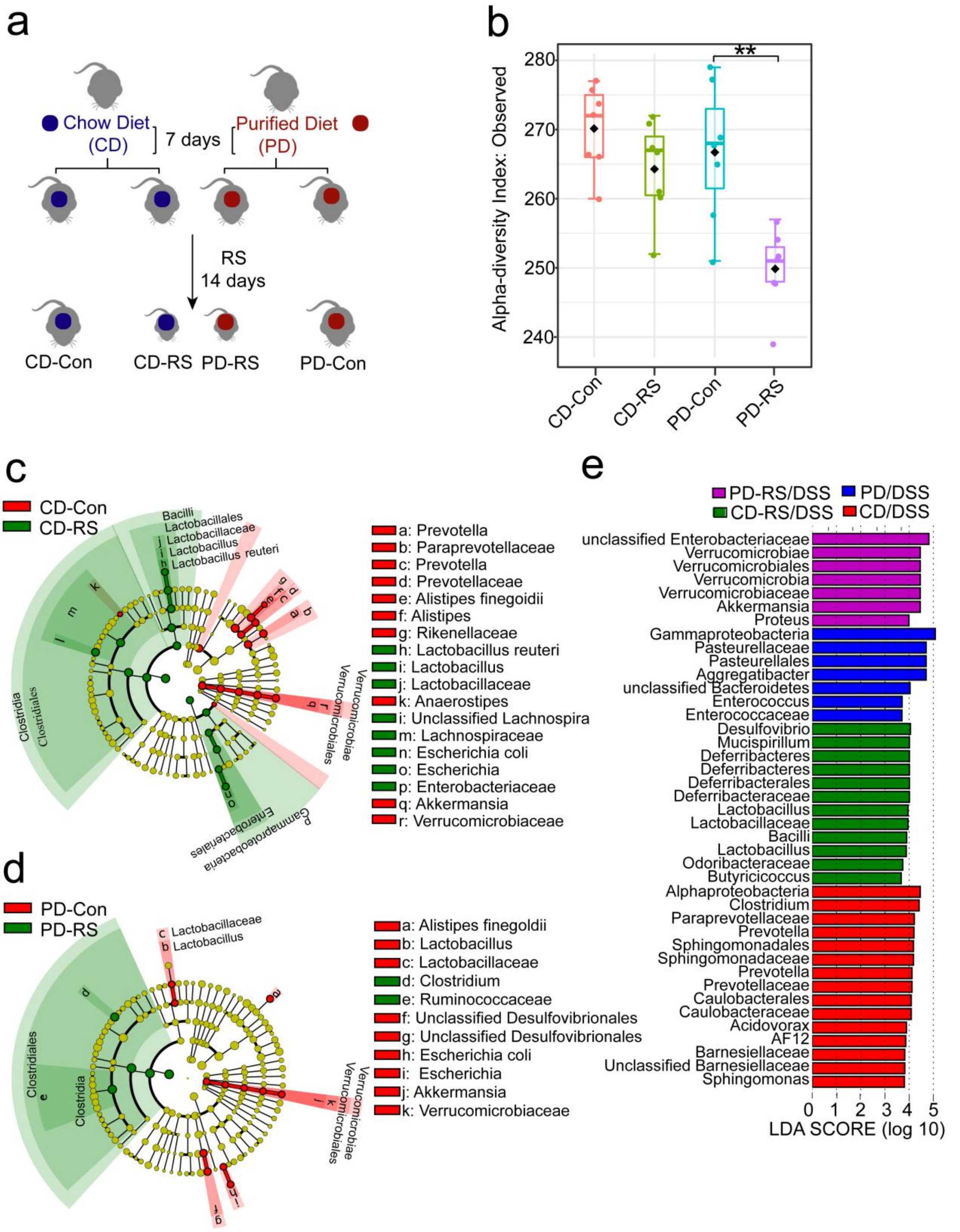

Supplementary Fig.3 Dietary pattern modulates stress-induced gut microbiota remodeling in mice

(**a**) Schematic of the experimental design. Mice were placed on standard chow (CD-Con/CD-RS) or purified diet (PD-Con/PD-RS), and subjected to 14 days of restraint

stress (RS).

(**b**) Box plots comparing the alpha diversity of the fecal microbiota as measured by number of observed species. Boxes show the medians and the interquartile ranges (IQRs), the whiskers denote the lowest and highest values that were within 1.5 times the IQR from the 25% and 75% quartiles, and outliers are shown as individual points. Statistical analysis was performed by Kruskal-Wallis test for n=7, $^{**}p < 0.01$.

(**c,d**) LDA effect size (LEfSe) cladogram of taxonomic data from 16S rRNA sequence analysis of fecal samples. Comparison was made between control (Con, red) and restraint stress (RS, green) mice fed with chow diet (**c**) or purified diet (**d**). The cladograms show the taxonomic levels represented by rings with phyla at the innermost ring and genera (identified in the legend) at the outermost ring, and each circle is a member within that level. Taxa at each level are shaded (red and green) according to the group in which it is most abundant ($p < 0.05$; LDA score 2.0).

(**e**) Linear discriminative analysis (LDA) score of differentially expressed bacteria obtained from LEfSe analysis of fecal microbial abundance from colitis mice. Genus with Kruskal-Wallis test ≤ 0.05, as well as LDA >3 is listed.

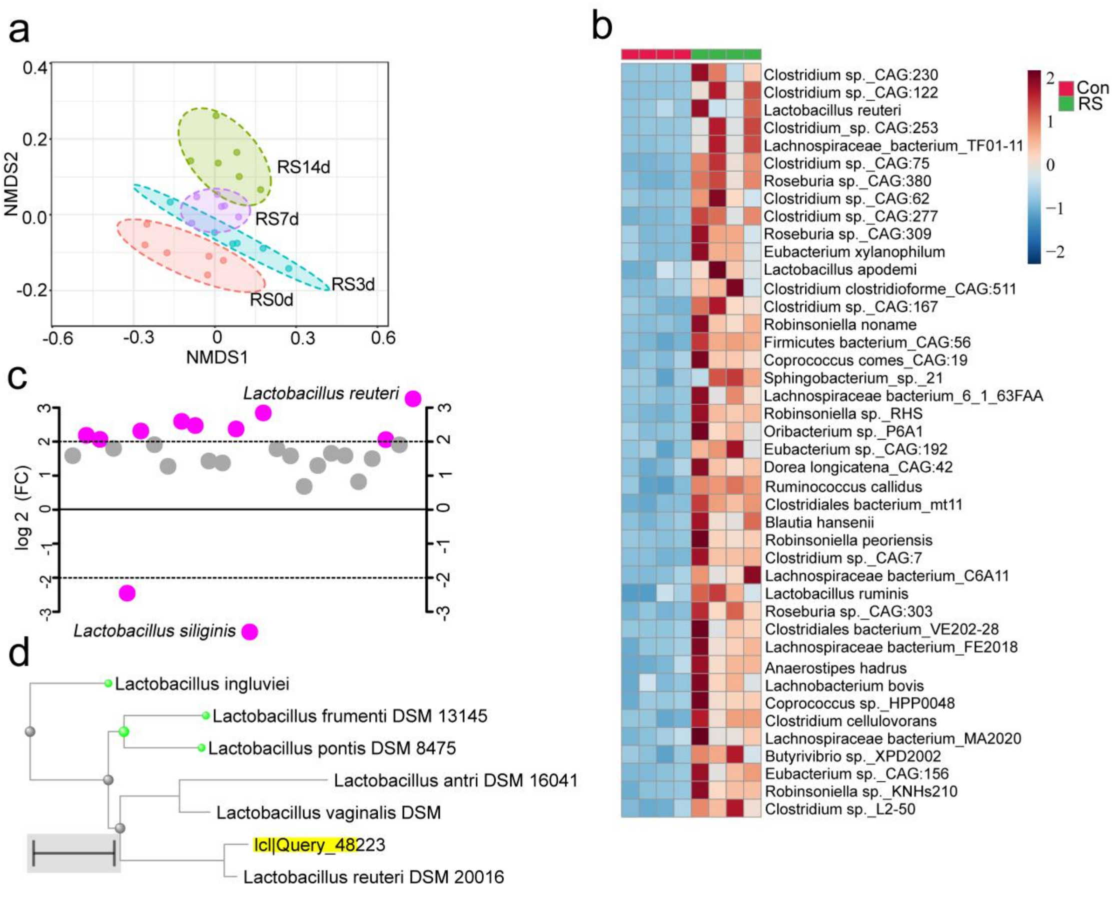

Supplementary Fig.4 Identification of a diet-sensitive *Lactobaccilus reuteri* strain from the fecal microbiota of stressed mice

(**a**) Nonmetric multi-dimensional scaling (NMDS) plot showing time-dependent changes of fecal microbial composition of CD-fed mice during the 14-day restraint stress.

(**b**) Heatmap showing stress-induced changes in relative abundance of the 43 most abundant species.

(**c**) Comparison of log2-fold changed abundances of *Lactobacillus spp.* species that are significantly altered after restraint stress. Points in red represent species for which a more than 4-fold increase/decrease in abundance was detected.

(**d**) Genomic sequence alignment and evolutionary analysis of the isolated *Lactobacillus* strain from mice. The evolutionary history was inferred using the Neighbor-Joining method. The query sequence (lclIQuery_48223, highlighted) is aligned with the reference strain *Lactobacillus reuteri* DSM 20016.

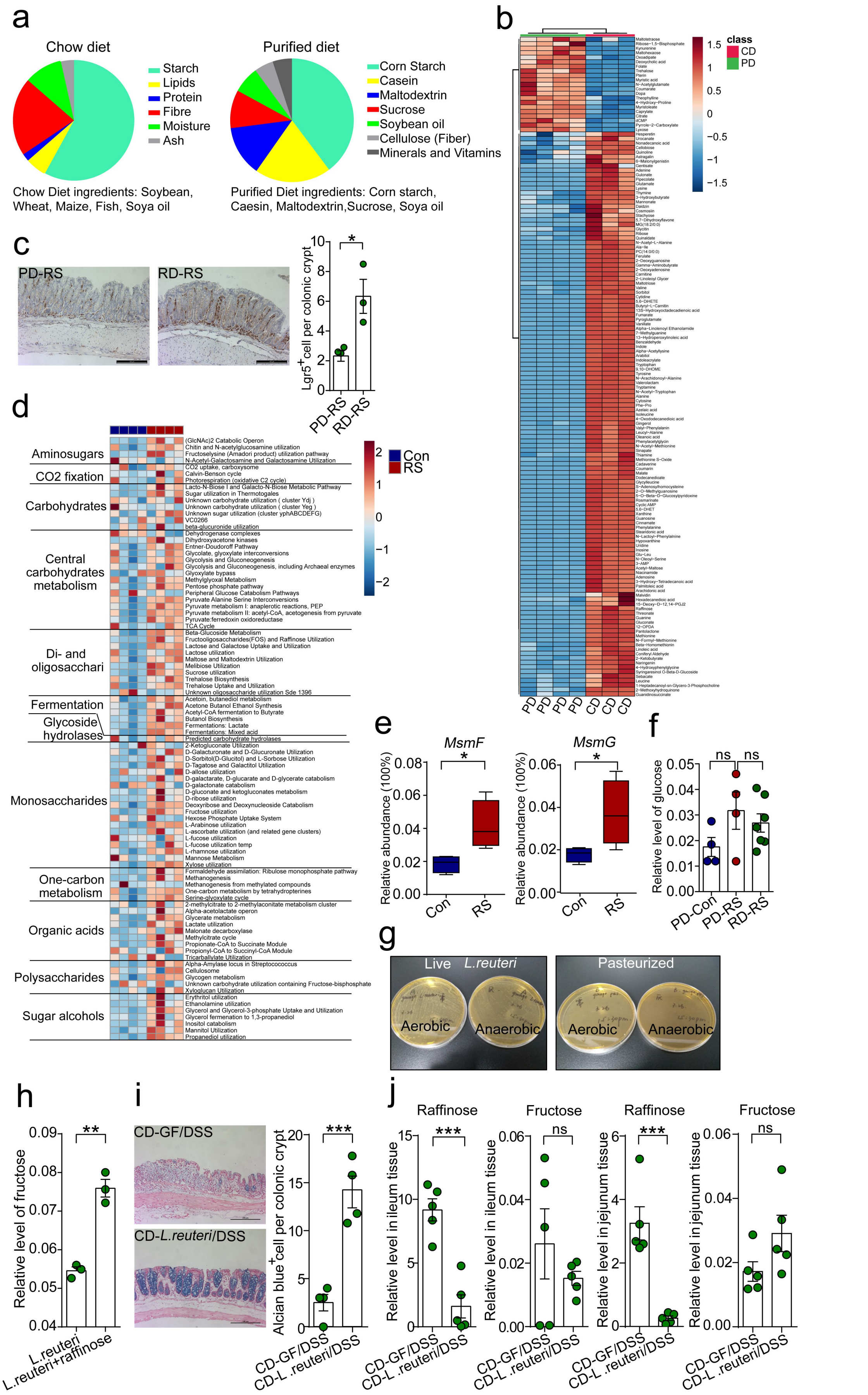
a
Chow diet
Starch
Lipids
Protein
Fibre
Moisture
Ash
Chow Diet ingredients: Soybean, Wheat, Maize, Fish, Soya oil
Purified diet
Corn Starch
Casein
Maltodextrin
Sucrose
Soybean oil
Cellulose (Fiber)
Minerals and Vitamins
Purified Diet ingredients: Corn starch, Caesin, Maltodextrin,Sucrose, Soya oil
b
class
CD
PD
PD PD PD PD CD CD CD
c
PD-RS
RD-RS
Lgr5+cell per colonic crypt
PD-RS RD-RS
d
Con
RS
Aminosugars
CO2 fixation
Carbohydrates
Central carbohydrates metabolism
Di- and oligosacchari
Fermentation
Glycoside hydrolases
Monosaccharides
One-carbon metabolism
Organic acids
Polysaccharides
Sugar alcohols
e
MsmF
MsmG
Relative abundance (100%)
Con RS
f
Relative level of glucose
ns ns
PD-Con PD-RS RD-RS
g
Live L.reuteri
Pasteurized
Aerobic
Anaerobic
h
Relative level of fructose
L.reuteri
L.reuteri+raffinose
i
CD-GF/DSS
CD-L.reuteri/DSS
Alcian blue+cell per colonic crypt
j
Raffinose
Fructose
Relative level in ileum tissue
Relative level in jejunum tissue
CD-GF/DSS
CD-L .reuteri/DSS

Supplementary Fig.5 Bacterial metabolism of dietary raffinose to fructose preserves ISC proliferation

(**a**) Pie chart showing the major nutritional composition and ingredients of CD versus PD.

(**b**) Heatmap depicting the ingredients differentially detected between CD and PD.

(**c**) Representative Lgr5 staining images and quantification of positive cells in colonic crypts from mice fed purified diet (PD) or raffinose-supplemented purified diet (RD) during 14-day restraint stress. Scale bar: 100 μm. Data are represented as Mean ± SEM for n=3 biologically independent samples (p=0.03 by unpaired two-tailed student's t-test).

(**d**) Metabolic pathway changes of gut microbiota induced by stress as predicted by SEED analysis of the metagenomic data.

(**e**) Box plot showing relative abundance of raffinose transporter *MsmF* and *MsmG* in the metagenomic data. Box limits are the 10th and 90th percentiles, center lines are median, and the whiskers are the minimal and maximal values (*$p < 0.05$ by Wilcoxon *rank-sum* test for n=4).

(**f**) Relative level of glucose in the ileum of mice fed with purified diet (PD) or purified diet supplemented with raffinose (RD). Data are represented as Mean ± SEM for PD group (n=4) and RD group (n=7); ns, no significance by one-way ANOVA.

(**g**) Representative images of *L. reuteri* colonies by culture from the feces of *L.reuteri* inoculated GF mice.

(**h**) Relative level of fructose after *L. reuteri DSM20016* incubation with or without raffinose (10 mM) for 24 hr. Data are represented as Mean ± SEM (p=0.001 by unpaired two-tailed student's t-test for n=3).

(**i**) Alcian blue (AB) mucin staining images and quantification of colonic goblet cells per crypt (n=4). Scale bar: 100 μm. Data are represented as Mean ± SEM for n=4 biologically independent samples (p=0.0007 by unpaired student's t-test).

(**j**) Relative level of raffinose and fructose in the ileum and jejunum section of GF or *L. reuteri*-colonized mice after DSS challenge. Data are represented as Mean ± SEM for n=5. $p = 0.0003$ and 0.0004 for raffinose in the ileum and jejunum by unpaired two-tailed student's t-test; ns, no significance.

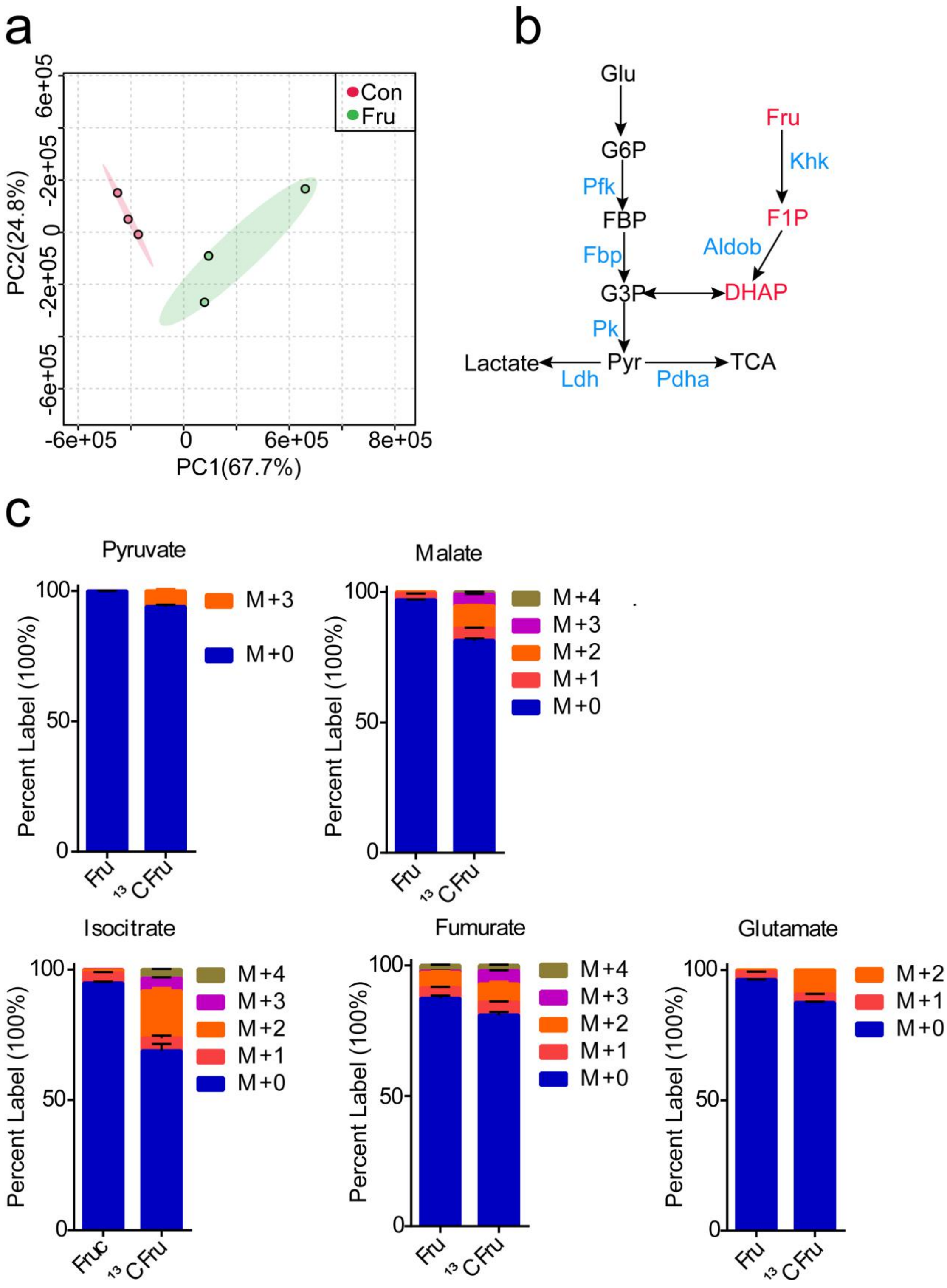

Supplementary Fig.6 Fructose induces metabolic programming required for organoid formation.

(**a**) PCA plot of metabolomic data from intestinal organoids, showing distinct metabolic signatures between vehicle and fructose-treated samples.

(**b**) Schematic showing fructose metabolism pathways and key enzymes previously reported in the liver. Khk, ketohexokinase; Pfk, phosphofructokinase; Fbp, fructose-bisphosphatase; Pk, pyruvate kinase; Aldob, aldolase b; Ldh2, lactate dehydrogenase 2; Pdha, pyruvate dehydrogenase complex.

(**c**) Percent labeling of the indicated metabolites following 6 hr incubation with 10 mM U-[^{13}C]-fructose (^{13}C Fru). Data are represented as Mean ± SEM for n=3.

Supplementary Table 1. Summary of the primer sequences for RT-PCR analysis of targeted genes of mouse origin.

Gene name	Forward primer (5'-3')	Reverse primer (5'-3')
Il6	TAGTCCTTCCTACCCCAATTTCC	TTGGTCCTTAGCCACTCCTTC
Il1β	GAAATGCCACCTTTTGACAGTG	TGGATGCTCTCATCAGGACAG
Tnfα	CCTCTCTCTAATCAGCCCTCTG	GAGGACCTGGGAGTAGATGAG
Mcp1	TAAAAACCTGGATCGGAACCAAA	GCATTAGCTTCAGATTTACGGGT
Gapdh	AGGTCGGTGTGAACGGATTTG	TGTAGACCATGTAGTTGAGGTCA
Aldoa	CGTGTGAATCCCTGCATTGG	CAGCCCCTGGGTAGTTGTC
Aldob	TGTCTGGAGGTATGAGTGAGG	CTGGGTTGCCTTCTTGTTTGC
Aldoc	CTCTACATCGCCAACCATGC	TCAAACATGGGAGGGCATCT
Eno1	TGCGTCCACTGGCATCTAC	CAGAGCAGGCGCAATAGTTTTA
Eno2	AGGTGGATCTCTATACTGCCAAA	GTCCCCATCCCTTAGTTCCAG
Eno3	CACAGCCAAGGGTCGATTCC	CCCAGGTATCGTGCTTTGTCT
Fbp1	GACTGGGGATCAAGTAAAGAAGC	AGGTAGCGTAGGACGACTTCA
Fbp2	AACTCGATGCTGACTGCCAT	TCCTGTCACATTCACGCTCC
Hk1	GGGAGAGGCCATTCTTCATC	CCAAAAATACGACCCCCTCT
Hk2	TGATCGCCTGCTTATTCACGG	AACCGCCTAGAAATCTCCAGA
Hk3	TGCTGCCCACATACGTGAG	GCCTGTCAGTGTTACCCACAA
ldh2	GGAGAAGCCGGTAGTGGAGAT	GGTCTGGTCACGGTTTGGAA
Pgk2	TTCTGCTAAGTTGACTCTGGACA	AGCCTTGATTCTCTGGTTGTTTG
Pfk	TAAAGTACACTTTGGCCCCC	AGCTATCGGTGTCCTGACCA
Pkm	GCCCGTGAAGATACCAACTC	CCATGAAGAGCATCATGCAG
Pkl	CATCCCTGCCTTGATCATCT	TATCGACTCAGAGCCTGTGG
Pdha1	TCATTTGCAAAATTACGGGA	AAGATGCTTGCCGCTGTATC
Pdhb	TCTGATGGTGCGCAGATTTA	AGTTGCCCATTCAAGACCA
