# Supplementary material for: A diet-microbial metabolism feedforward loop modulates intestinal stem cell renewal in the stressed gut
Source: Nat Commun. 2021 Jan 11;12:271. doi: 10.1038/s41467-020-20673-4 (PMC7801547; doi:10.1038/s41467-020-20673-4)
Supplement: Supplementary file 2 — Reporting Summary [file 41467_2020_20673_MOESM2_ESM.pdf]

nature research

Corresponding author(s): Haiping Hao, Xiao Zheng

Last updated by author(s): Dec 12, 2020

Reporting Summary

Nature Research wishes to improve the reproducibility of the work that we publish. This form provides structure for consistency and transparency in reporting. For further information on Nature Research policies, see our Editorial Policies and the Editorial Policy Checklist.

Statistics

For all statistical analyses, confirm that the following items are present in the figure legend, table legend, main text, or Methods section.

n/a	Confirmed	
☐	☒	The exact sample size (*n*) for each experimental group/condition, given as a discrete number and unit of measurement
☐	☒	A statement on whether measurements were taken from distinct samples or whether the same sample was measured repeatedly
☐	☒	The statistical test(s) used AND whether they are one- or two-sided<br>*Only common tests should be described solely by name; describe more complex techniques in the Methods section.*
☒	☐	A description of all covariates tested
☐	☒	A description of any assumptions or corrections, such as tests of normality and adjustment for multiple comparisons
☐	☒	A full description of the statistical parameters including central tendency (e.g. means) or other basic estimates (e.g. regression coefficient) AND variation (e.g. standard deviation) or associated estimates of uncertainty (e.g. confidence intervals)
☐	☒	For null hypothesis testing, the test statistic (e.g. *F*, *t*, *r*) with confidence intervals, effect sizes, degrees of freedom and *P* value noted<br>*Give P values as exact values whenever suitable.*
☒	☐	For Bayesian analysis, information on the choice of priors and Markov chain Monte Carlo settings
☒	☐	For hierarchical and complex designs, identification of the appropriate level for tests and full reporting of outcomes
☒	☐	Estimates of effect sizes (e.g. Cohen's *d*, Pearson's *r*), indicating how they were calculated

Our web collection on statistics for biologists contains articles on many of the points above.

Software and code

Policy information about availability of computer code

Data collection	Principal-component analysis, Random forest, LEfSe analysis of the microbiome data were conducted using the web-based tool MicrobiomeAnalyst 4.0 (www.microbiomeanalyst.ca); Metabolic pathway enrichment analysis of these identified metabolic biomarkers was carried out by MetaboAnalyst 3.0. (http://www.metaboanalyst.ca/faces/ModuleView.xhtml); Raw data of LC-MS analysis were obtained with MassLynx v4.1 and analyzed by Quanlynx v4.1 (Waters, Milford, MA).
Data analysis	The raw data for the 16S rRNA and metagenomic sequencing were analyzed with QIIME (version 4.2; http://drive5.com/uchime/uchime_download.html), UPARSE (version 7.1; http://drive5.com/uparse/) and BLASTP (Version 2.2.28+). Statistical analysis was performed using GraphPad Prism 6.0 (Graphpad Software, Inc., San Diego, CA, USA).

For manuscripts utilizing custom algorithms or software that are central to the research but not yet described in published literature, software must be made available to editors and reviewers. We strongly encourage code deposition in a community repository (e.g. GitHub). See the Nature Research guidelines for submitting code & software for further information.

Data

Policy information about availability of data

All manuscripts must include a data availability statement. This statement should provide the following information, where applicable:

- Accession codes, unique identifiers, or web links for publicly available datasets
- A list of figures that have associated raw data
- A description of any restrictions on data availability

Source data for Figs. 1-6 and Supplementary Figs. 1-6 are provided with the paper. Other data that support the findings of this study are available from the corresponding author (H.H.) upon reasonable request. The 16S rRNA and metagenomic sequencing dataset are available from the NCBI Sequence Read Archive (SRA) repository with the accession number: PRJNA675376 [https://www.ncbi.nlm.nih.gov/sra/?term=PRJNA675376], PRJNA675375 [https://www.ncbi.nlm.nih.gov/

sra/?term=PRJNA675375], PRJNA675371 [https://www.ncbi.nlm.nih.gov/sra/?term=PRJNA675371], PRJNA675369 [https://www.ncbi.nlm.nih.gov/sra/?term=PRJNA675369] and PRJNA675416 [https://www.ncbi.nlm.nih.gov/sra/?term=PRJNA675416]. Raw data for metabolomics are deposited to Metabolomics Workbench (https://www.metabolomicsworkbench.org/search/sitesearch.php) with the Project ID: PR001041.

Field-specific reporting

Please select the one below that is the best fit for your research. If you are not sure, read the appropriate sections before making your selection.

[x] Life sciences [ ] Behavioural & social sciences [ ] Ecological, evolutionary & environmental sciences

For a reference copy of the document with all sections, see nature.com/documents/nr-reporting-summary-flat.pdf

Life sciences study design

All studies must disclose on these points even when the disclosure is negative.

Sample size	Sample sizes were determined from previous experience with these types of experiments, which were sufficient to generate reproducible results with desirable significance (0.05) and power (>90%).
Data exclusions	No data were excluded from the analyses.
Replication	Experiments with DSS in SPF mice were repeated for 2-3 times with satisfactory reproducibility. In vitro experiments on organoids were replicated for at least twice with success.
Randomization	Allocation was random. Detailed definitions and descriptions were provided in the manuscript.
Blinding	Investigators were blinded to group allocation during data collection and analysis wherever possible. This was not possible during real-time treatment of live animals in mouse studies, as the treatment of each mouse would need to be known to the person handling the mice. Investigators were blinded during subsequent histological analyses.

Reporting for specific materials, systems and methods

We require information from authors about some types of materials, experimental systems and methods used in many studies. Here, indicate whether each material, system or method listed is relevant to your study. If you are not sure if a list item applies to your research, read the appropriate section before selecting a response.

Materials & experimental systems

n/a	Involved in the study	
[ ]	[x]	Antibodies
[x]	[ ]	Eukaryotic cell lines
[x]	[ ]	Palaeontology and archaeology
[ ]	[x]	Animals and other organisms
[x]	[ ]	Human research participants
[x]	[ ]	Clinical data
[x]	[ ]	Dual use research of concern

Methods

n/a	Involved in the study	
[x]	[ ]	ChIP-seq
[x]	[ ]	Flow cytometry
[x]	[ ]	MRI-based neuroimaging

Antibodies

Antibodies used	Primary antibody against Olfm4, Lgr5 and Lysozyme were purchased from Cell Signaling Technology (Cat# 39141), Abcam (Cat# ab273092) and Abcam (Cat# ab105808), respectively.
Validation	The primary antibodies were fully validated for immunofluorescence (IF) staining by the manufacturer with validation results available on the official website. It was further validated in our IF assays on tissue sections of mice origin.

Animals and other organisms

Policy information about studies involving animals; ARRIVE guidelines recommended for reporting animal research

Laboratory animals	Six-week-old female SPF Balb/c mice were used for most of the animal study. All the mice were housed under controlled conditions with a 12 h light/dark cycle, 20-22 °C and 45 ± 5% humidity. For germ-free mice studies, both male and female mice 7 to 9 weeks of age were used and randomly assigned to different experimental groups.
Wild animals	None.
Field-collected samples	None.

Ethics oversight	The animal studies were approved by the Animal Ethics Committee of China Pharmaceutical University.

Note that full information on the approval of the study protocol must also be provided in the manuscript.
